# Transcriptome analysis suggested that lncRNAs regulate rapeseed seedlings in responding to drought stress by coordinating the phytohormone signal transduction pathways

**DOI:** 10.1186/s12864-024-10624-4

**Published:** 2024-07-19

**Authors:** Xiaoyu Tan, Weihua Long, Ni Ma, Shifei Sang, Shanya Cai

**Affiliations:** 1 School of Agronomy and Horticulture, Jiangsu Vocational College of Agriculture and Forestry, Zhenjiang, China; 2 School of Rural Revitalization, Jiangsu Open University, Nanjing, China; 3 Key Laboratory of Biology and Genetic Improvement of Oil Crops, Ministry of Agriculture and Rural Affairs, Oilcrops Research Institute of the Chinese Academy of Agricultural, Wuhan, China; 4 College of Life Sciences, Henan Normal University, Xinxiang, China

**Keywords:** *Brassica napus* L., Water stress, Rehydration, lncRNA, Signal transduction

## Abstract

The growth, yield, and seed quality of rapeseed are negatively affected by drought stress. Therefore, it is of great value to understand the molecular mechanism behind this phenomenon. In a previous study, long non-coding RNAs (lncRNAs) were found to play a key role in the response of rapeseed seedlings to drought stress. However, many questions remained unanswered. This study was the first to investigate the expression profile of lncRNAs not only under control and drought treatment, but also under the rehydration treatment. A total of 381 differentially expressed lncRNA and 10,253 differentially expressed mRNAs were identified in the comparison between drought stress and control condition. In the transition from drought stress to rehydration, 477 differentially expressed lncRNAs and 12,543 differentially expressed mRNAs were detected. After identifying the differentially expressed (DE) lncRNAs, the comprehensive lncRNAs-engaged network with the co-expressed mRNAs in leaves under control, drought and rehydration was investigated. The Kyoto Encyclopedia of Genes and Genomes (KEGG) enrichment analysis of co-expressed mRNAs identified the most significant pathways related with plant hormones (expecially abscisic acid, auxin, cytokinins, and gibberellins) in the signal transduction. The genes, co-expressed with the most-enriched DE-lncRNAs, were considered as the most effective candidates in the water-loss and water-recovery processes, including protein phosphatase 2 C (PP2C), ABRE-binding factors (ABFs), and SMALL AUXIN UP-REGULATED RNAs (SAURs). In summary, these analyses clearly demonstrated that DE-lncRNAs can act as a regulatory hub in plant-water interaction by controlling phytohormone signaling pathways and provided an alternative way to explore the complex mechanisms of drought tolerance in rapeseed.

**Supplementary Information:**

The online version contains supplementary material available at 10.1186/s12864-024-10624-4.

## Introduction

Drought stress is a significant threat to global agricultural production [1]. Plants respond to drought stress through morphological changes visually, such as stomata closure, leaf area reduction, and root growth promotion, as well as physiological, biochemical, and molecular mechanisms internally, including osmotic regulation, antioxidant synthesis, and upregulation of related gene expression [2]. Drought stress can negatively impact plant growth, photosynthesis, respiration, and organ development, ultimately affect the crop yield and the quality of agriculture product [3]. Drought resistance/tolerance mechanisms involve complex biological processes, such as gene expression, signal transduction regulation, and cellular metabolic rates with key pathways including the ABA-dependent/independent pathway and the abscisic acid signaling system [4–6]. Under drought stress, stress signals are transmitted through various transduction pathways in plants, and some important stress signals can regulate stress-inducing genes. The expression and regulation of these related functional genes can change the morphological structure of plants or the physiological and biochemical indexes of cells to improve the drought resistance of plants [7].

In recent years, there has been a growing interest in studying the post-transcriptional level regulation to understand anti-stress mechanisms, with a focus on the function of non-coding RNAs [8, 9]. Long non-coding RNAs (lncRNAs) are RNA molecules longer than 200 nucleotides that do not encode proteins, which have been shown to play a role in plant resistance to drought stress [10]. In *Arabidopsis*, the *DROUGHT INDUCED lncRNA (DRIR)* regulates plant response to drought stress by modulating the expression of genes involved in ABA signaling, water transport, and other stress-reducing processes [11]. In rice, the lncRNA MSTRG.28732.3 works with miR171 to the target genes involved in chlorophyll membrane synthesis (i.e., *Os02g0662700*, *Os02g0663100*, and *Os06g0105350*) in plants [12]. In cotton, a large number of lncRNAs associate with ethylene, auxin, gibberellins, and cytokinins under drought stress [13]. In cassava leaves and roots, 124 drought-responsive lncRNAs regulate the expression of their neighboring genes involved in hormone metabolism, transcriptional RNA regulation, and receptor kinase signaling [14]. Two drought resistance-related lncRNAs were identified in tetraploid cassava, which affect the stomatal density [15]. In tomato, some drought stress-related lncRNAs were found to promote the expression of target genes specifically enriched in response to the stimuli, signaling, and transporter activity [16]. In Tibetan wild barley, ten lncRNAs were exclusively induced by drought stress and the lncRNA-mRNA interaction-based analysis identified the potential regulator, a serine/threonine-protein kinase SMG1 [17]. However, the comprehensive survey of lncRNAs involved in drought-responsive regulation in rapeseed are still lacking; and it is unclear whether lncRNA-mRNA co-expression networks are involved in the response to drought stress.

Rapeseed (*Brassica napus* L., AACC, 2n = 38) is an important oilseed crop cultivated worldwide for producing edible oil, animal feed, and biodiesel. However, rapeseed is reported to be very sensitive to water deficits during the germination and seedling growth stages [18]. Drought stress not only reduces the yield of rapeseed but also limits its adaptability. Therefore, drought has become one of the major limiting abiotic factors hindering the production and promotion of rapeseed, especially in China. In a previous study, we conducted a genome-wide lncRNAs analysis between drought-tolerant and drought-sensitive genotypes and analyzed the possible lncRNAs’ function between different genetic background under drought stress, providing some clues for understanding the drought-resistant mechanism [19]. In this study, we aim to explore the lncRNA and mRNA expression divergence profiles of a drought-tolerant germplasm under control, drought plus rehydration treatments, so as to identify the key lncRNAs involved in the different water-supply environments.

## Results

### Phenotype of rapeseed seedlings under different treatments

**Fig. 1:**
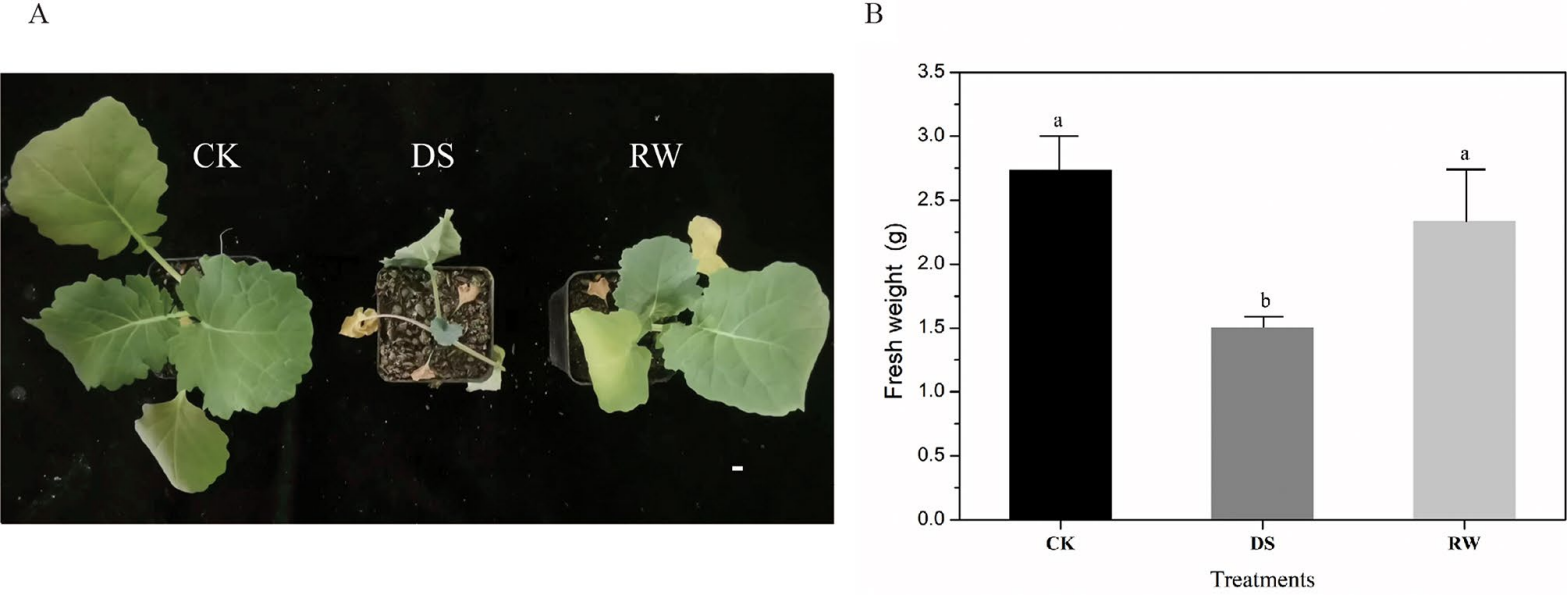
Phenotype of seedlings under different treatments. (**A**) Phenotypes of seedlings under CK, DS, and RW treatments. Bar = 1 cm. (**B**) Comparisons of plant height among the treatments. (**C**) Comparisons of fresh weight among the treatments. The experiments were repeated three times and vertical bars indicated standard errors. CK = control; DS = drought stress; RW = re-watering. The same letter indicates no significant difference and the different letters indicate a significant difference at a significance level of 5%

The seedlings under control (CK), drought stress (DS), and re-watering (RW) treatments exhibited different morphologies. Compared to CK, seedlings under DS showed typical dehydration symptoms, such as leaf wilting and growth retardation (Fig. 1A); however, after rehydration, seedlings appeared to recover from drought and leaves became strong and healthy again (Fig. 1A). Compared to CK, seedlings under DS had a significant reduction in fresh weight. The seedlings under RW were observed to have larger leaves. Similar with CK, the fresh weight of seedlings under RW was significantly higher than that under DS (Fig. 1B). Thus, rehydration alleviated the symptoms of drought stress in rapeseed seedlings.

### Hormone contents in the leaves under different treatments

**Fig. 2:**
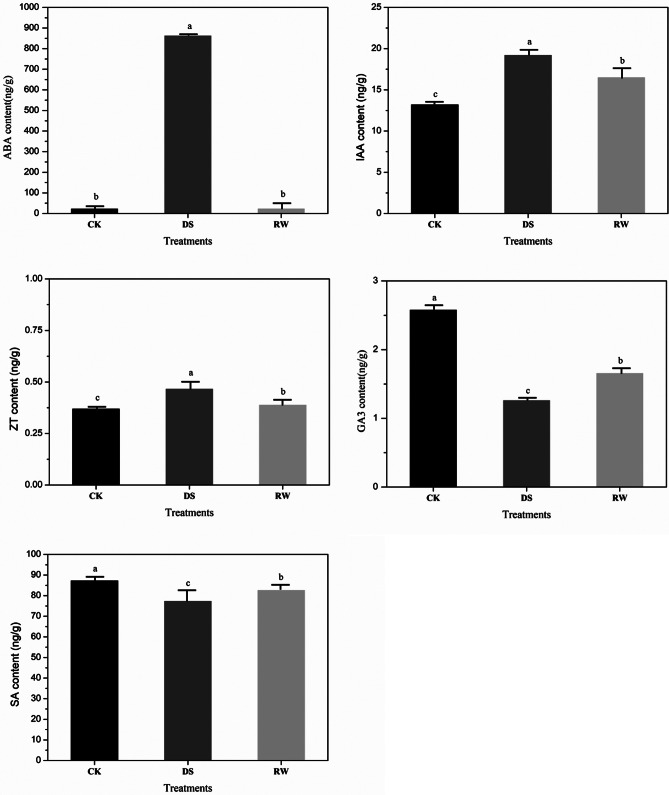
Contents of three hormones in seedling leaves under CK, DS, and RW treatments. ABA = abscisic acid; IAA = auxin; ZT = zeatin; GA3 = gibberellic acid 3; SA = salicylic acid. Experiments were repeated three times, and vertical bars indicate standard errors. CK = control; DS = drought stress; RW = re-watering. The same letter indicates no significant difference and the different letters indicate a significant difference at a significance level of 5%

Studies have shown that phytohormones such as auxin, abscisic acid, cytokinin, gibberellins, and salicylic acid could regulate plant tolerance to drought stress [20, 21]. Five hormones (auxin (IAA), abscisic acid (ABA), zeatin (ZT), gibberellic acid 3 (GA3) and salicylic acid (SA)) were measured in the leaves under the CK, DS and RW treatments (Fig. 2). Compared with that under CK, the ABA content increased 37-fold under the DS treatment, the IAA content increased by 45.38%, the ZT content increased by 26.42%, the GA3 content decreased by 50.04%; moreover, the SA content decreased by 11.41%. Following the RW treatment, the ABA content nearly reached the CK level again, the IAA content was 1.25 times higher than the CK, the ZT content was restored to 1.05 times of the CK level, and the GA3 content was restored to 64.25% of the CK level, while the SA content was restored to 94.67% of the CK level. Therefore, drought stress and rehydration treatment affected the metabolism of these hormones.

### RNA sequencing and data analysis

RNAs were successfully extracted from all samples (three biological replicates for each treatment) and were qualified before performing RNA sequencing (Supplementary Table 1). Clean reads were obtained by removing low-quality reads from the RNA-seq data, following standard protocols. Quality and GC-content data were then calculated from the clean data to assess the quality of the sequencing data (Supplementary Table 2). The clean datasets were then mapped to the *B. napus* reference genome (http://www.brassicagenome.net/databases.php). All the parameters showed the reliability of the RNA-sequencing data, and the datasets could be incredibly powerful for subsequent analysis.

### Validation of sequencing data by quantitative real-time PCR (qPCR) analysis

**Fig. 3:**
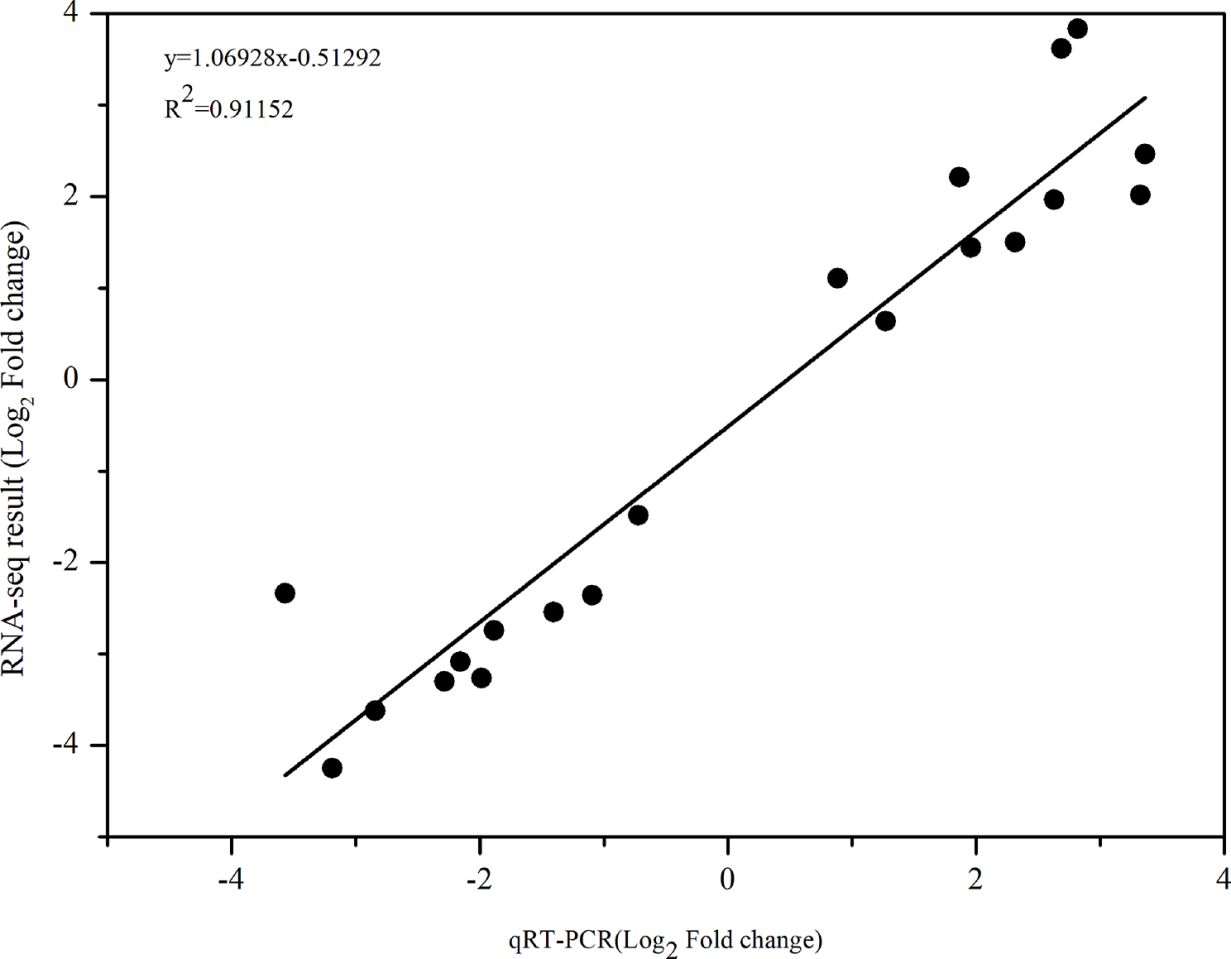
Validation of the expression levels of lncRNAs using the real-time quantitative polymerase chain reaction (RT-qPCR). The x-axis indicates the log_2_(Fold change) as measured by RT-qPCR. The y-axis indicates the log_2_(Fold change) as measured by RNA sequencing (RNA-seq). The squared of the Pearson’s correlation coefficient of relative expression measured by RNA-seq and RT-qPCR was 0.91152

Ten differentially expressed DE-lncRNAs were randomly selected for qRT-PCR verification. The *R*^*2*^ value (*R*^*2*^ > 0.9) indicated a significant correlation between the expression levels of the DE-lncRNAs quantified by FPKM and qRT-PCR results (Fig. 3). For example, the relative expression of XLOC_000799 decreased in DS vs. CK, while increased in RW vs. DS, which was consistent with the RNA-seq result (Supplementary Table 3). The real-time PCR results confirmed the expression patterns derived from transcriptome sequencing, indicating the reliability of the RNA-Seq analysis results.

### Differentially expressed lncRNAs (DE-lncRNAs) and mRNAs (DE-mRNAs) under different treatment

**Fig. 4:**
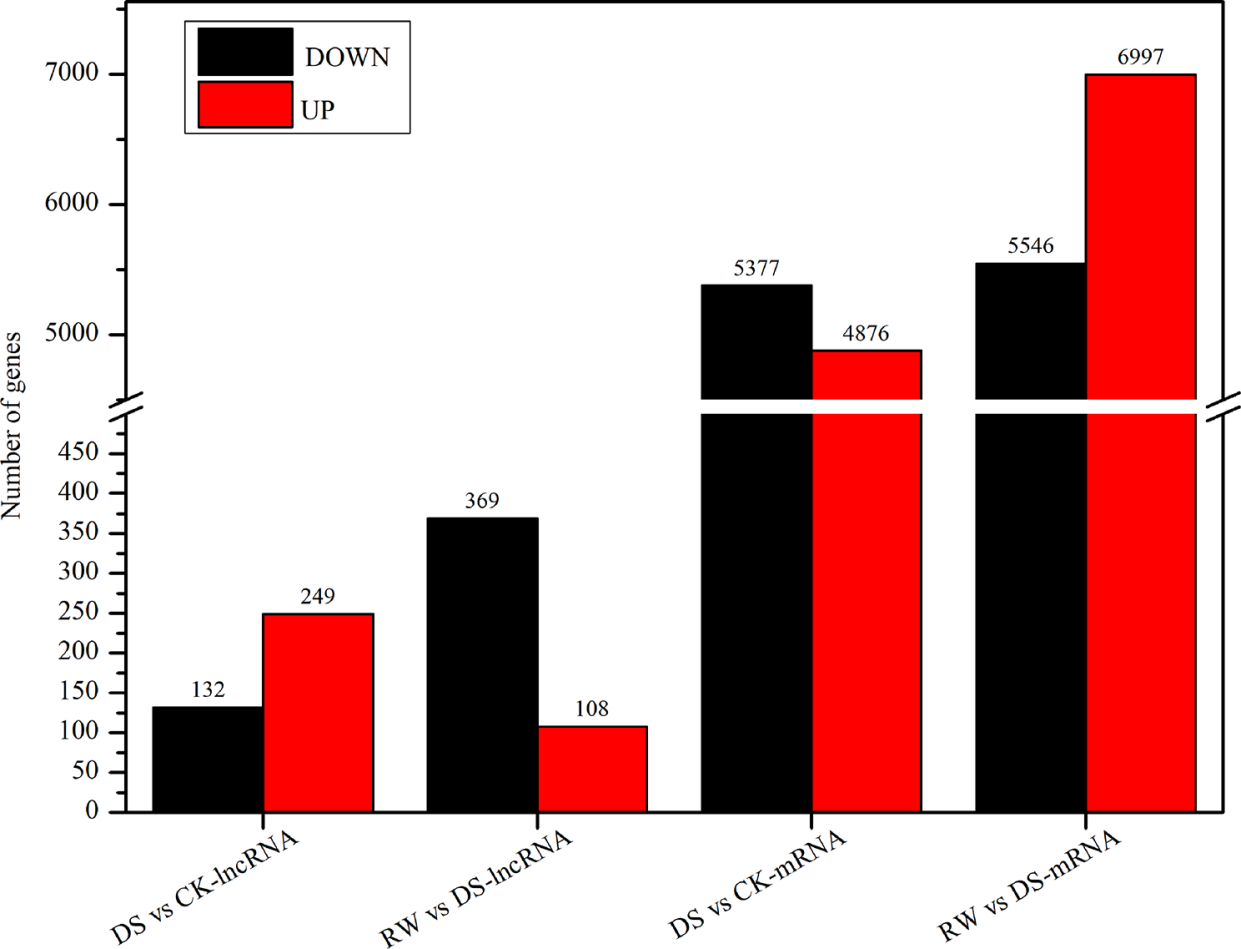
The numbers of DE-lncRNAs and DE-mRNAs in two comparison groups

By comparing the amount of each lncRNA and mRNA sequence from leaves in CK, DS, and RW treatments, DE-lncRNAs and DE-mRNAs were screened with a *q*-value threshold of < 0.05 by performing comparisons between DS vs. CK and RW vs. DS. A total of 381 DE-lncRNAs (132 down-regulated, 249 up-regulated) and 10,253 DE-mRNAs (5,377 down-regulated, 4,876 up-regulated) were identified in DS vs. CK, while 477 DE-lncRNAs (369 down-regulated, 108 up-regulated) and 12,543 DE-mRNAs (5,546 down-regulated, 6,997 up-regulated) were identified in RW vs. DS (Fig. 4). The data revealed three main findings: (1) In general, there were more DE-lncRNAs and DE-mRNAs in RW vs. DS than in DS vs. CK; (2) there were more up-regulated DE-lncRNAs in DS vs. CK than down-regulated DE-lncRNAs, while the opposite was observed in RW vs. DS; and (3) there were more down-regulated DE-mRNAs in DS vs. CK than up-regulated DE-mRNAs, while the opposite was observed in RW vs. DS.

### Function and pathway analysis of DE-lncRNAs based on lncRNA-mRNA co-expression network in each comparison

**Fig. 5:**
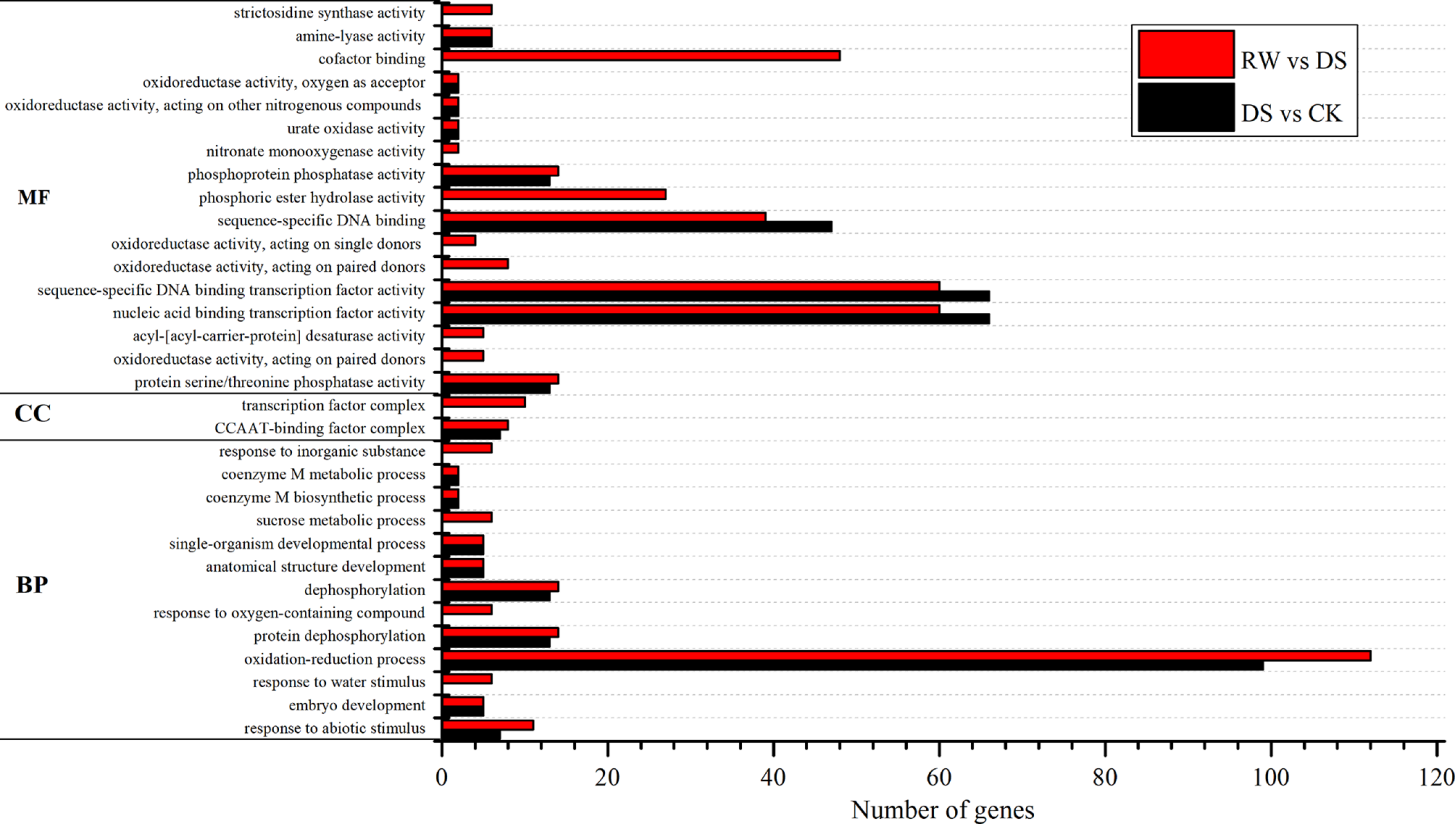
Gene Ontology (GO) classifications of the co-expressed mRNAs of the differentially expressed lncRNAs. The mRNAs co-expressed with lncRNAs were divided into three main categories by GO analysis: biological process, molecular functions, and cellular components. The x-axis indicates the number of genes in a sub-category, and the y-axis indicates the sub-categories

We used lncRNA-mRNA relationship pairs to construct an interactive network to characterize the roles and functions of DE-lncRNAs. In DS vs. CK, there were 3,493 lncRNA-mRNA pairs (including 1,423 mRNAs and 102 lncRNAs) (Supplementary Table 4). Similarly, there were 5,175 lncRNA-mRNA pairs in RW vs. DS (including 1,481 mRNAs and 145 lncRNAs) (Supplementary Table 4). Gene Ontology (GO) analysis was applied on the target mRNAs to analyze their biological processes (BPs), cellular components (CCs), and molecular functions (MFs) (Fig. 5).

In DS vs. CK, 20 GO terms were significantly enriched, including oxidation-reduction process (GO:0055114), protein dephosphorylation (GO:0006470), dephosphorylation (GO:0016311), response to abiotic stimulus (GO:0009628), and embryo development (GO:0009790) for BPs. For MFs, nucleic acid binding transcription factor activity (GO:0001071), sequence-specific DNA binding transcription factor activity (GO:0003700), sequence-specific DNA binding (GO:0043565), protein serine/threonine phosphatase activity (GO:0004722), and phosphoprotein phosphatase activity (GO:0004721) were the most important significantly enriched GO terms. The GO term of CCAAT-binding factor complex (GO:0016602) was the most significant term for CCs.

In RW vs. DS, 32 GO terms were significantly enriched, including oxidation-reduction process (GO:0055114), protein dephosphorylation (GO:0006470), dephosphorylation (GO:0016311), response to abiotic stimulus (GO:0009628), and response to water stimulus (GO:0009415) for BPs. With respect to MPs, nucleic acid binding transcription factor activity (GO:0001071), sequence-specific DNA binding transcription factor activity (GO:0003700), cofactor binding (GO:0048037), sequence-specific DNA binding (GO:0043565), and phosphoric ester hydrolase activity (GO:0042578), were the dominant groups. For CCs, transcription factor complex (GO:0005667) and CCAAT-binding factor complex (GO:0016602) were the most dominant groups.

**Fig. 6:**
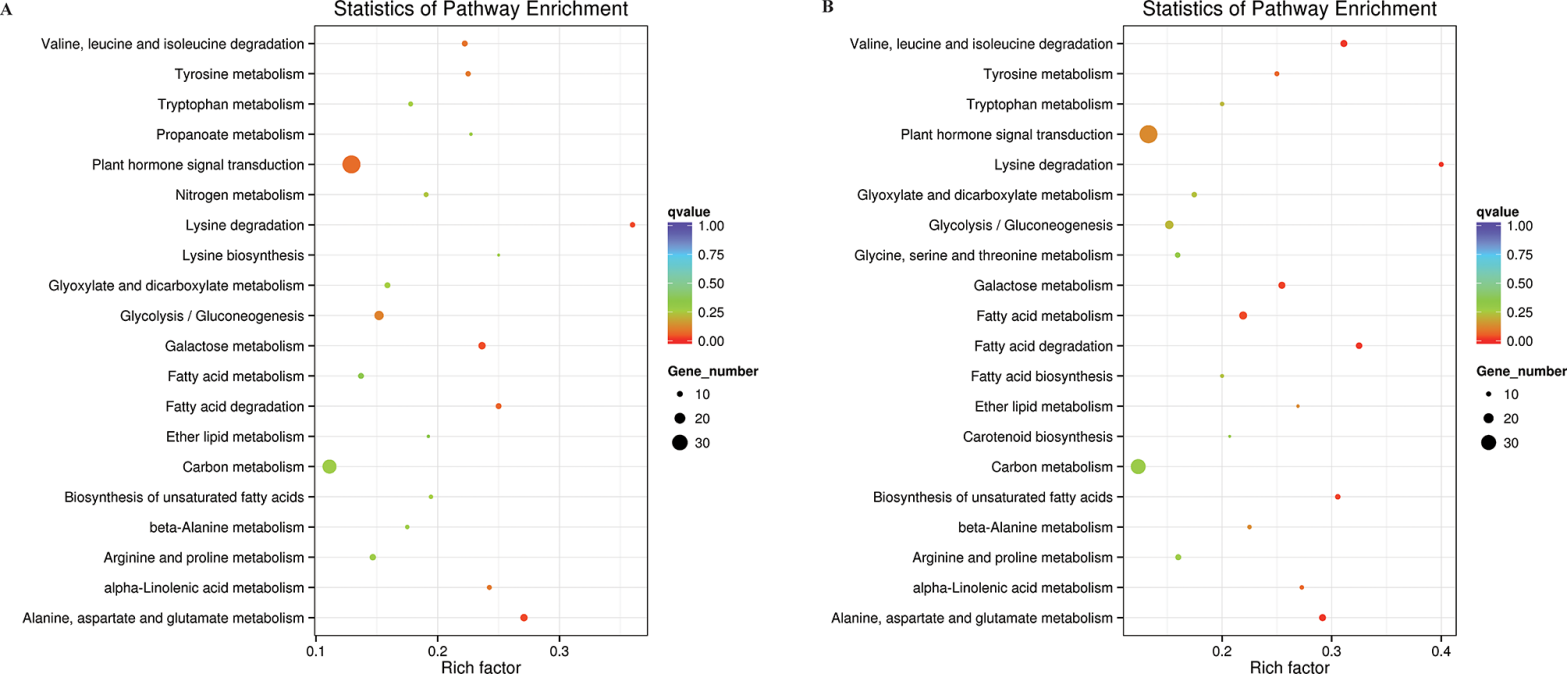
KEGG pathway analysis. Top 20 pathways for the co-expressed mRNAs of the differentially expressed lncRNAs. The y-axis corresponds to the KEGG pathway with a *q*-value ≤ 0.05, and the x-axis shows the enrichment ratio between the number of differentially expressed genes and all UniGenes enriched in a particular pathway. The color of the dot represents *q* value, and the size of the dot represents the number of differentially expressed genes mapped to the reference pathways. (**A**) KEGG pathway classification of the mRNAs co-expressed with DE-lncRNAs in DS vs. CK. (**B**) KEGG pathway classification of the mRNAs co-expressed with DE-lncRNAs in RW vs. DS

There were 18 KEGG pathways identified as significant in DS vs. CK and RW vs. DS groups. In the DS vs. CK group, the target mRNA genes that co-expressed with DE-lncRNAs were significantly enriched in plant hormone signal transduction (ko04075), carbon metabolism (ko01200), glycolysis/gluconeogenesis (ko00010), alanine, aspartate, and glutamate metabolism (ko00250), and galactose metabolism (ko00052) (Fig. 6A). In the RW vs. DS group, several metabolic and signal transduction pathways, such as plant hormone signal transduction (ko04075), carbon metabolism (ko01200), glycolysis/gluconeogenesis (ko00010), fatty acid metabolism (ko01212), as well as valine, leucine, and isoleucine degradation (ko00280) were significantly enriched and they had a large number of target genes (Fig. 6B).

### Deep analysis on the key DE-lncRNAs which continuously function during the water loss and water-retaining process

**Fig. 7:**
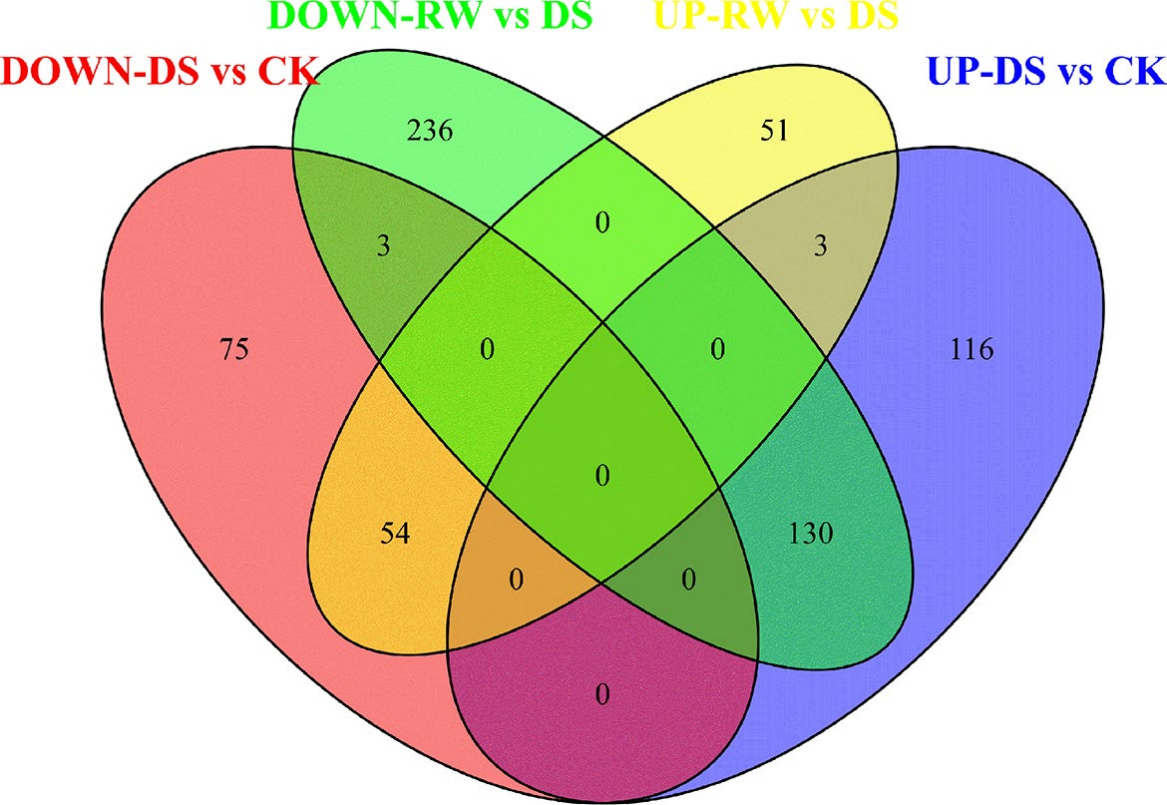
Venn diagram showing the number of unique and common DE-lncRNAs in two comparison groups

The Venn diagram in Fig. 7 showed the numbers of independent and overlapping DE-lncRNAs for each comparison. A total of 190 DE-lncRNAs were identified in two comparisons, of which three were up-regulated and three were down-regulated in DS vs. CK and RW vs. DS. Since dehydration and rehydration have opposite effects, the regulation direction of lncRNAs should be also opposite in these two treatments. Therefore, the remaining 184 DE-lncRNAs were the focus of subsequent analysis. Among these, 54 DE-lncRNAs were down-regulated in DS vs. CK but up-regulated in RW vs. DS, and 11 of these lncRNAs were found to have 37 partner mRNAs. Similarly, 130 DE-lncRNAs were up-regulated in DS vs. CK but down-regulated in RW vs. DS, and 56 of these lncRNAs were found to have 998 partner mRNAs. These partner-mRNAs were used for subsequent Go analysis and KEGG enrichment. Based on the significance threshold of *p* < 0.05, these co-expressed target mRNA genes were assigned to 20 significant terms (Supplementary Table 5). In the BP category, the top five terms were oxidation-reduction process (GO:0055114), protein dephosphorylation (GO:0006470), dephosphorylation (GO:0016311), response to abiotic stimulus (GO:0009628), and embryo development (GO:0009790). In the MF category, the top five terms were nucleic acid binding transcription factor activity (GO:0001071), sequence-specific DNA binding transcription factor activity (GO:0003700), sequence-specific DNA binding (GO:0043565), phosphoprotein phosphatase activity (GO:0004721), and protein serine/threonine phosphatase activity (GO:0004722). Notably, CCAAT-binding factor complex (GO:0016602) was the only enriched term in the CCs category.

KEGG pathway analysis was separately conducted in two comparison groups to understand the biological pathways in water-deficient or water-sufficient environments (Supplementary Table 6). Three pathways, including plant hormone signal transduction (35 genes in DS vs. CK and 36 genes in RW vs. DS), carbon metabolism (27 genes in DS vs. CK and 30 genes in RW vs. DS), and glycolysis/gluconeogenesis (17 genes in DS vs. CK and 17 genes in RW vs. DS), were significantly enriched in both comparison groups with the largest number of genes.

Because too many GO analysis results were enriched in the comparison, we used the KEGG results to focus on the major melatonin-related pathways and pivotal genes, which would emphasize the main melatonin function.

Due to the excessive GO analysis results in the two comparison series (DS vs. CK and RW vs. DS), we prioritized the most important drought-related pathways and key genes based on the KEGG results. Interestingly, KEGG analysis revealed that most genes in the plant hormone signal transduction pathway were enriched. To further investigate the mechanism of lncRNAs functioning under drought stress, we focused on analyzing the lncRNAs co-expressed with genes involved in plant signal transduction pathways.

### Analysis of DE-lncRNAs involved in plant hormone signal transduction pathways

**Fig. 8:**
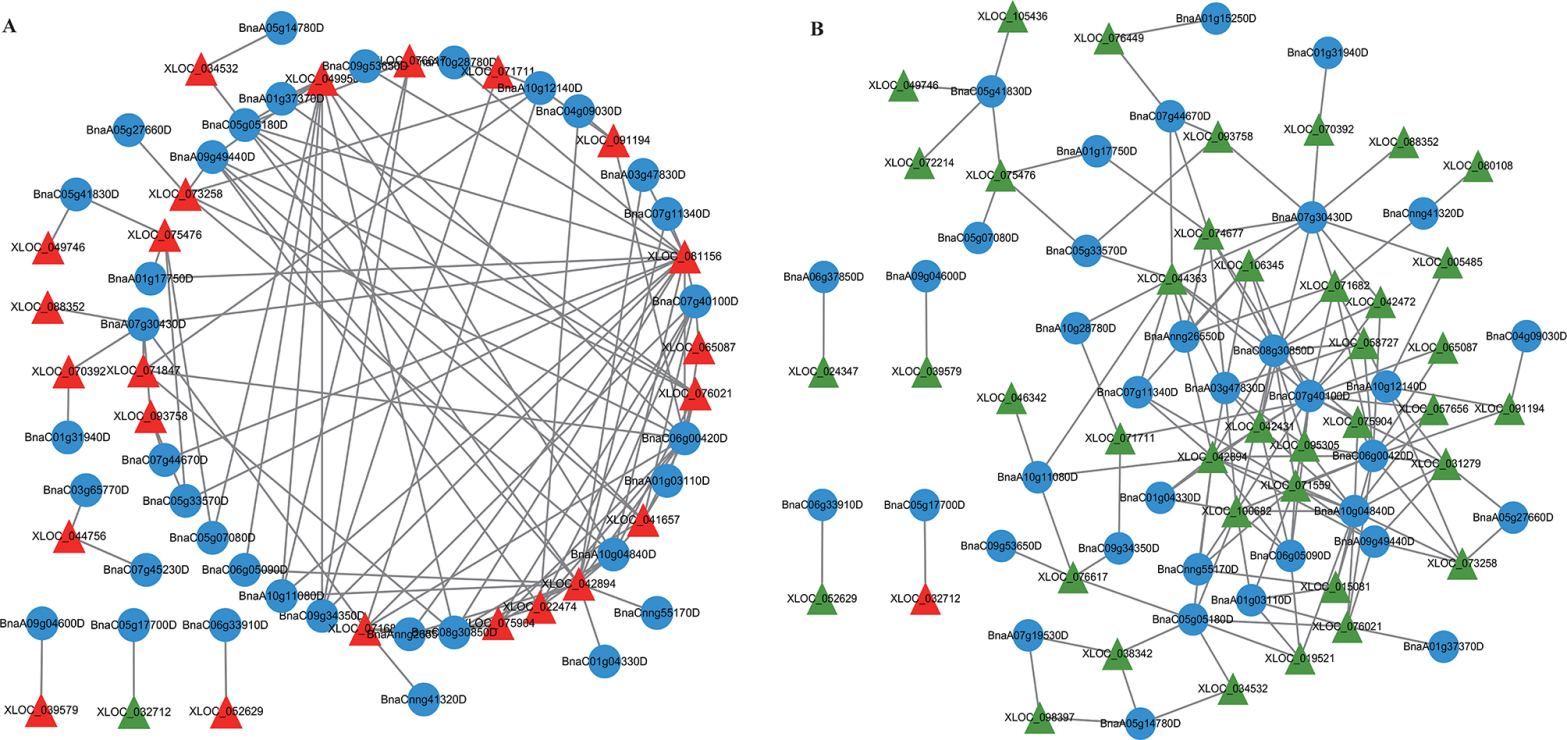
LncRNA-mRNA-network analysis of the plant hormone signal transduction. The circle and rectangle nodes represent lncRNAs and protein-coding genes, respectively. The up-regulated and down-regulated nodes are separately colored in red and green. Edges show regulatory interactions among nodes. (**A**) 24 DE-lncRNAs interacted with 35 mRNAs in the meaningful “plant hormone signal transduction” in DS vs. CK. (**B**) 41 DE-lncRNAs interacted with 36 mRNAs in the meaningful “plant hormone signal transduction” in RW vs. DS

**Fig. 9:**
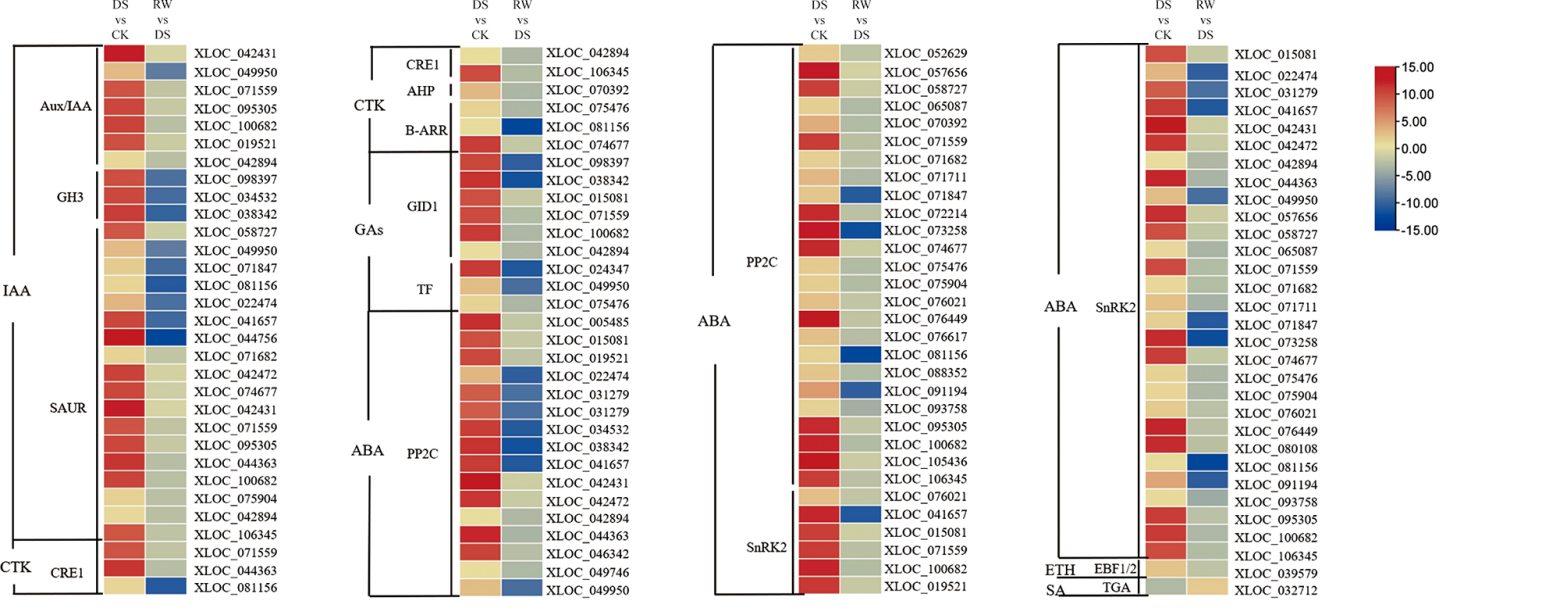
Expression levels of transcripts involved in phytohormone signal transduction pathways in DS vs. CK and RW vs. DS

**Fig. 10:**
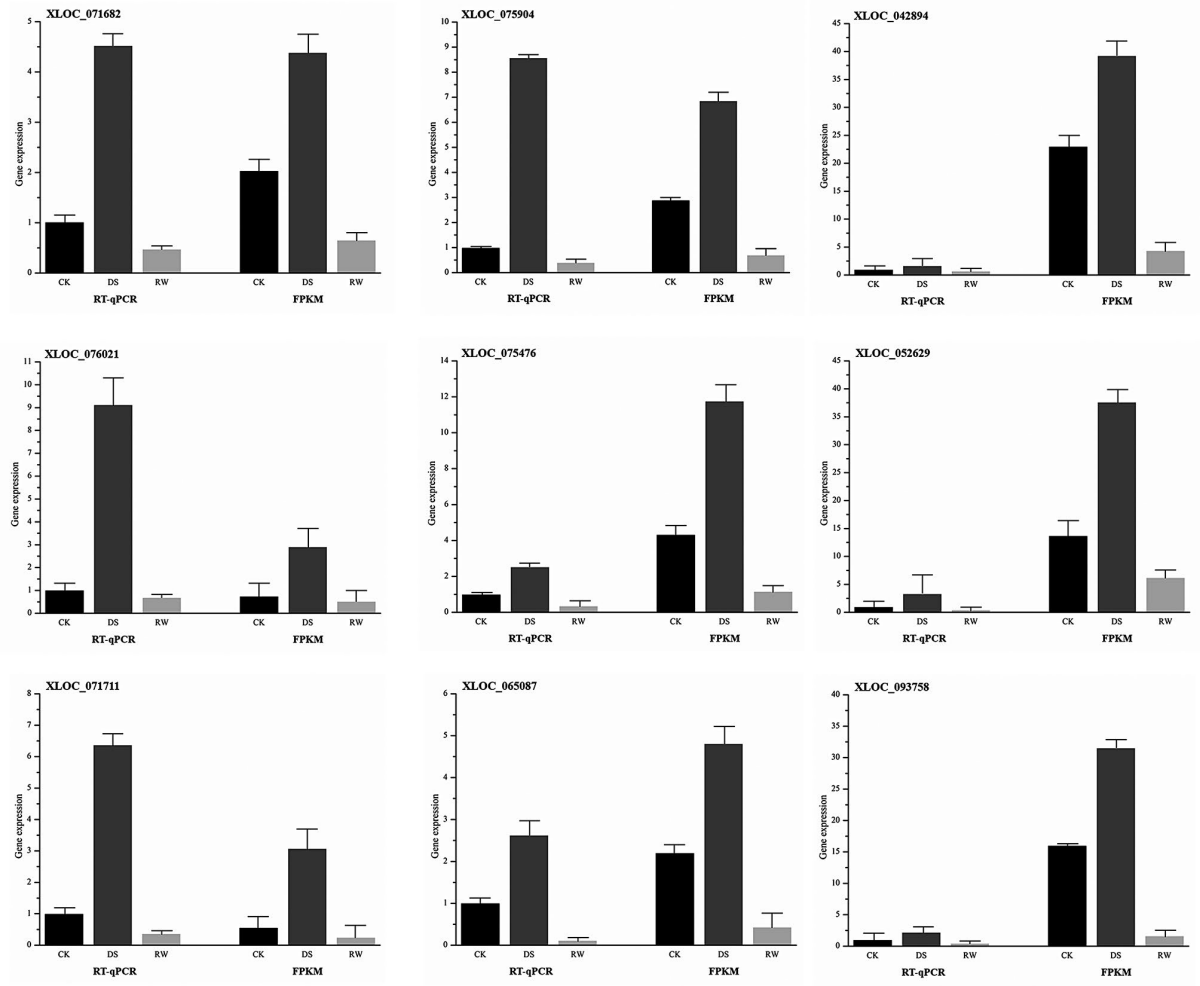
Confirmation of the expression patterns of key lncRNAs under drought and rehydration conditions using quantitative RT-PCR

Since over 15% of co-expressed mRNAs of DE-lncRNAs were enriched in the pathway of plant hormone signal transduction in both comparisons, this pathway was selected as the focus. In DS vs. CK, the co-expression network under this pathway contained 108 matched lncRNA-mRNA pairs, including 24 lncRNAs and 35 mRNAs (Fig. 8A and Supplementary Table 7). The target genes of 24 DE-lncRNAs consisted of 1 down-regulated mRNA and 34 up-regulated mRNAs. In RW vs. DS, the co-expression network of plant hormone signal transduction contained 157 matched lncRNA-mRNA pairs, including 41 lncRNAs and 36 mRNAs (Fig. 8B and Supplementary Table 7). The target genes of the 41 DE-lncRNAs consisted of 35 down-regulated mRNAs and 1 up-regulated mRNA. As shown in Fig. 9, the expression trends or levels of these lncRNAs co-expressed with auxin, cytokinins, gibberellins, abscisic acid, ethylene, and salicylic acid signal transduction genes were completely different under drought and rewater treatment. Within this pathway, the target genes involved in abscisic acid (ABA) signal transduction were dominant (65.7% and 66.7% in the two comparisons). The genes with the highest co-expression frequency included type 2 C protein phosphatases (PP2Cs) and abscisic acid-responsive transcription factors (ABFs) in the ABA metabolism pathway, as well as small auxin upregulated RNAs (SAURs) in the IAA metabolism pathway. The expression levels of lncRNAs co-expressed with some genes involved in plant hormone signal transduction (PP2Cs, ABFs, and SAURs) were measured using qRT-PCR, and the results of qRT-PCR were consistent with the RNA-seq data, indicating that these lncRNAs were key genes in response to drought and rehydration (Fig. 10).

## Discussion

Abiotic stress seriously affects agricultural development, and lncRNAs have been shown to take important parts in regulate crops’ response to the environments at the molecular level [22]. Water supply is a vital environmental factor in agriculture, and excessive water or insufficient water (drought) supply can both have a negative impact on crops [23]. Studies have shown that lncRNAs function under drought stress, but their molecular mechanism is not yet well understood. In previous studies, we only compared rehydration treatment with drought stress and identified lncRNAs related to rehydration as involved in plant hormone signal transduction. So, what pathways and functions do lncRNAs induced by drought stress participate in? In this study, libraries of both lncRNA and mRNA were constructed under normal conditions (CK), drought stress (DS), and re-watering (RW). The contents of five phytohormones were measured under different treatment conditions. Our data supported the comparisons of the whole process, from normal to drought and then to rehydration, at the physiological level and the molecular level. We presented a comprehensive analysis to uncover the crucial role of important lncRNAs in the process of water loss and rehydration.

### Phenotypic changes of rapeseed seedlings during water-losing and rehydration

The phenotypic and physical parameters indicated significant differences in the growth status of rapeseed seedlings under various water supply conditions. The DS treatment decreased the seedling weight significantly and the RW treatment recovered it to a similar level as CK, indicating that the temporary damage from drought is reversible and repairable in rapeseed seedlings, which is according with Xu et al. [24]. However, the seedling phenotype uder RW was not consistant with that under DS, implying the molecular-level activities happened during the water loss and water supply enviroments.

### Genes related with the phytohormones signal transduction during drought stress

Although 190 co-expressing DE-lncRNAs were identified as being related to many biological processes, the number of lncRNAs involved in plant hormone signal transduction was the largest. In this pathway, target genes of DE-lncRNAs were mostly involved in abscisic acid, auxin, cytokinin, and gibberellins signaling pathways in both comparison groups.

**Abscisic acid (ABA)** is a key phytohormone that is essential in regulating various growth and metabolic processes in response to biotic and abiotic stresses, especially drought stress [25]. The increase in ABA concentration in the soil solution under drought stress stimulates ABA signaling in roots and stems, regulating the water status of plants [26, 27]. The ABA signaling system includes the ABA receptor proteins PYR/PYL/RCAR, the positive regulator SnRK2 (*SNF1* associated protein kinase 2), and the negative regulator 2 C protein phosphatases (PP2C), as well as their downstream targets. These three components-PYR/PYL/RCAR, SnRK2, and PP2C-combine as a dual negative regulatory system to regulate ABA signaling and its downstream responses [28, 29].

However, some studies have shown that subfamily A PP2Cs in *Arabidopsis* and other plants negatively regulates ABA and stress signaling pathways [30–32]. On the other hand, *BdPP2CA6*, a subfamily A PP2C in *Brachypodium distachyon*, was found to be a positive regulator of ABA and stress signaling pathways [33]. Similarly, the expression of *SiPP2C10* in foxtail millet has been upregulated under drought stress conditions [34]. This finding was consistent with the results of our study, leading us to speculate that these lncRNAs co-expressed with PP2C may be closely related to drought resistance genes.

In this study, we found that both the two core components of the ABA signal transduction pathway and the downstream central ABF component were regulated by drought stress. When rapeseed seedlings were exposed to drought stress during early growth stages, ABA content was significantly increased. The expression of lncRNAs co-expressed with PP2C, SnRK2 and ABF coding genes was upregulated by high concentration of ABA, resulting in enhanced ABA signal, which may increase the inhibition of ABA on the growth of rapeseed seedlings (Supplementary Figure S1A). After rehydration, ABA content almost recovered to the control level, and the expression of lncRNAs co-expressed with PP2C, SnRK2, and ABF was down-regulated, resulting in the weakening of the ABA signal, which alleviated the inhibitory effect of ABA on the growth of rapeseed seedlings (Supplementary Figure S1B). The analysis of the lncRNA-mRNA co-expression network showed that the genes encoding PP2C, SnRK2, and ABF had similar expression patterns during both the drought and rewatering processes. However, the lncRNAs co-expressed with these genes had not only different expression patterns, but also different numbers, revealing that rehydration is not just a simple process of restoring drought stress, but also involves lncRNAs co-expression with mRNAs in the regulation of a diverse range of recovery processes.

**Auxin (IAA)** is a major regulatory signal for plant growth and development and negatively regulates plant drought resistance [35, 36]. The IAA early response genes, such as *GH3*, *Aux/IAA*, and *SAUR*, play a role in the IAA signaling pathway [37, 38]. The Aux/IAA protein functions as a transcriptional inhibitor in the IAA signal transduction pathway [39]. *GH3* encodes auxin-conjugating enzymes that are involved in stress responses by controlling the levels of active auxin through negative feedback [40]. The *SAUR39* gene of rice acts as a negative regulator of auxin synthesis and transport [41]. The co-expression network of lncRNA-mRNA, *Aux/IAA*, *GH3*, and *SAUR* genes in the auxin signal transduction pathway was analyzed in response to drought stress and rehydration.

IAA content increased significantly after drought stress, the signaling pathway was activated, and lncRNAs co-expressed with *Aux/IAA*, *GH3*, and *SAUR* were upregulated, indicating that stress restricted the growth of rape seedlings by inhibiting IAA signal transduction (Supplementary Figure S2A). After rehydration, IAA content decreased, resulting in decreased expression of lncRNAs co-expressed with *Aux/IAA*, *GH3*, and *SAUR* genes, which accelerated the vegetative growth by stimulating tissue elongation and cell expansion (Supplementary Figure S2B).

**Cytokinins (CTKs)** not only manage plant growth and development but also play a role in mediating plant tolerance to drought stress [42]. The accumulation of CTKs can have both positive and negative effects on plant drought resistance [43]. *Arabidopsis* seedlings use a two-component signaling system (TCS) to regulate cytokinin signaling, which consists of sensor histidine kinases (AHKs), histidine phosphate transfer proteins (AHPs), and response regulators (ARRs) [44]. Cytokinin Response 1 (CRE1) was found to negatively regulate osmotic pressure in the presence of CTKs [45]. In *Arabidopsis*, AHP2, AHP3, and AHP5 serve as redundant negative regulators in response to drought stress [46]. The expression B-type cytokinin response regulators *ARR1*, *ARR10*, and *ARR12* in *Arabidopsis* were inhibited by drought stress, indicating that they negatively regulate plant drought tolerance [47].

The content of zeatin, the main natural active component of CTKs, increased under drought stress, which caused the up-regulation of lncRNAs co-expressed with CRE1, AHP and B-ARR. Compared with drought treatment, zeatin content decreased after rehydration treatment, which led to downregulation of lncRNAs co-expressed with CRE1, AHP and B-ARR (Supplementary Figure S3). Results of the study indicated that drought stress inhibited cytokinin signal transduction, leading to reduced growth of rapeseed seedlings. Conversely, after rehydration, an increase in cytokinin signal transduction resulted in improved growth of the seedlings. Additionally, CRE1, AHP, and B-ARR were found to play a role as negative regulators in the growth of rapeseed seedlings under drought stress.

Interestingly, the co-expression of lncRNA-mRNA revealed that while CRE1 (BnaC07g11340D), AHP (BnaC01g31940D), and B-ARR (BnaA01g17750D) were involved in cytokinin signal transduction during both drought and rehydration, and the lncRNAs co-expressed with these genes in the two treatments differed. It suggested that rehydration was not merely a simple drought recovery process, but that lncRNAs also played a role in this process.

**Gibberellins (GAs)** not only regulate seed germination, stem elongation, and flower development but also participate in the regulation of abiotic stress [48, 49]. There are many kinds of gibberellins, among which GA1, GA3, GA4, and GA7 have the highest biological activity [49]. The synthesis of gibberellin is inhibited by drought stress [50]. Research has shown that reducing GAs levels can improve drought resistance and help plants overcome water deficits through drought avoidance [50, 51]. The sensing of gibberellins (GAs) is mediated by GID1 (GA-INSENSITIVE DWARF1), a receptor like a hormone-sensitive lipase [52]. When GA binds to GID1s, it stimulates interaction between GID1s and the growth-inhibiting DELLA [53].

In our experiment, the content of GA3 in leaves decreased significantly under drought stress. After re-watering treatment, the content of GA3 increased significantly compared with that after drought treatment, although the level of GA3 was not up to the level of control. During drought stress, the expression of the lncRNA (XLOC_042894) that co-expressed with BnaCnng55170D (encoding GID1) was up-regulated (Supplementary Figure S4A). After rehydration, the expression of lncRNAs (XLOC_098397, XLOC_038342, XLOC_015081, XLOC_071559, XLOC_100682, and XLOC_042894) that co-expressed with BnaA07g19530D and BnaCnng55170D (encoding GID1) were down-regulated (Supplementary Figure S4B). The results showed that drought stress reduced the content of GA3 in rapeseed seedlings, and GID1 did not bind with low concentrations of GA3, which made the DELLA protein bind to the gibberellin response gene and inhibit its activity, thus inhibiting the growth of rapeseed seedlings. Rehydration inhibited the repression of DELLA on GA signaling, resulting in improved growth of rapeseed seedlings.

In conclusion, the number of lncRNAs co expressed with the genes encoding PP2C and ABF is the highest in the ABA signaling pathway. Moreover, compared to drought stress, rehydration treatment mobilized more lncRNAs to participate in ABA, IAA, CTKs, and GAs signal transduction, helping rapeseed seedlings recover growth.

### The proposal model showing LncRNAs’ function in response to drought stress and rehydration in rapeseed seedings

**Fig. 11:**
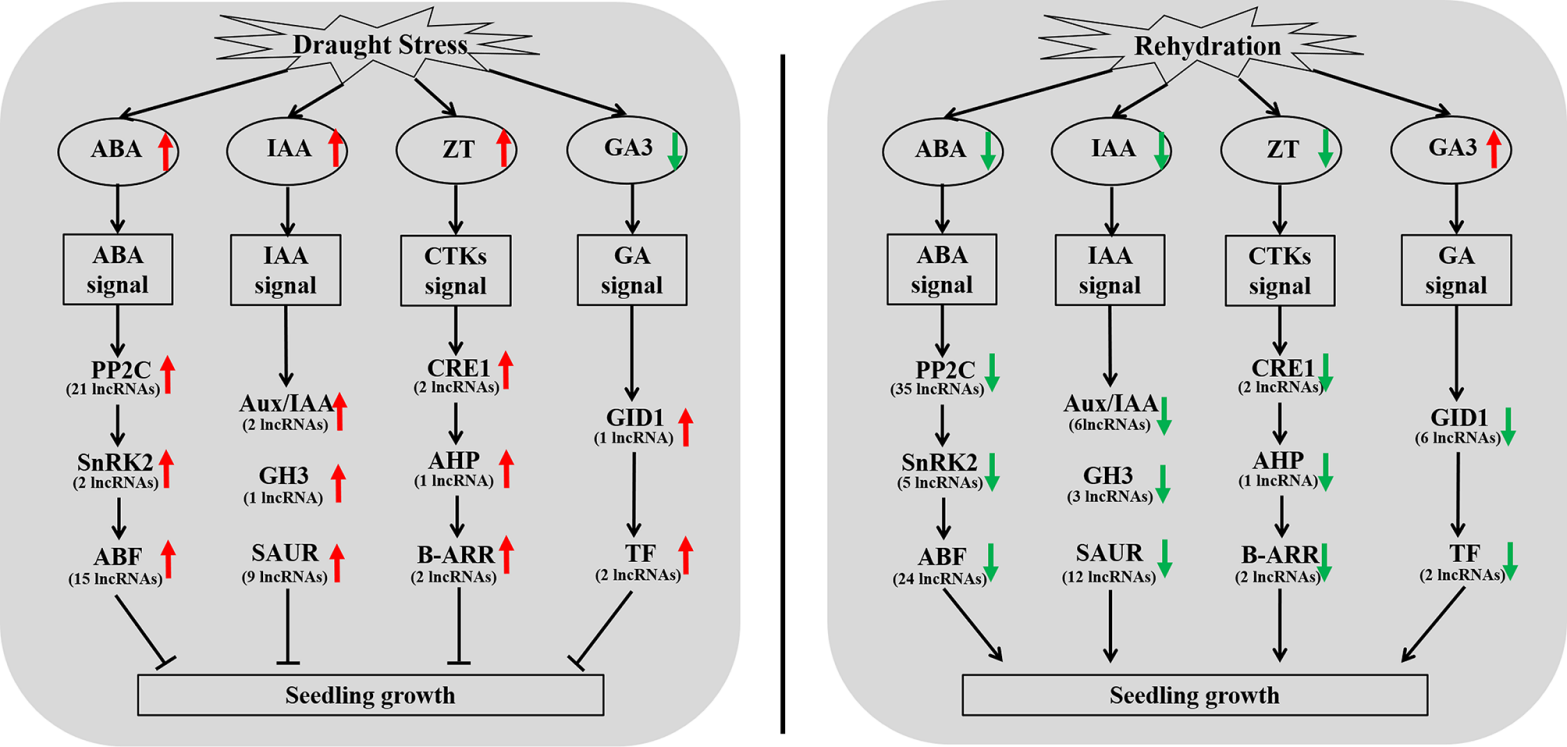
The model for the function of lncRNAs in response to drought stress in rapeseed seedlings. The up-pointing red arrows mean that the candidate lncRNAs are up-regulated; the down-pointing green arrows mean that the candidate lncRNAs are down-regulated. The main affected pathways were shown in the boxes. The number of important lncRNAs affecting the corresponding pathway is indicated in brackets. ABA = abscisic acid; IAA = auxin; CTKs = cytokinins; GAs = gibberellins; ZT = zeatin; GA3 = gibberellic acid 3

By focusing on DE-lncRNAs, we proposed possible mechanisms. Under drought stress, certain specific lncRNAs co-expressed with genes were responsible for the corresponding hormone’s signal transduction, regulating the expression of downstream genes and affecting seedlings growth. For instance, under drought stress, 21, 2, and 15 lncRNAs were up-regulated and co-expressed with PP2C, SnRK2, and ABF, respectively, promoting ABA signal transduction and intensifying the inhibitory effect of ABA on the rapeseed seedlings. After rehydration, 35, 5, and 24 lncRNAs co-expressed with PP2C, SnRK2, and ABF were down-regulated, releasing the inhibition, and allowing the seedlings to continue growing. It should be noted that ABA, IAA, and GAs are the major hormones involved in directing seedling growth (Fig. 11).

## Materials and methods

### Plant materials, growth conditions, and treatments

A drought-tolerant *B. napus* germplasm Q2, obtained from the Oil Crops Research Institute (OCRI) of the Chinese Academy of Agricultural Sciences (CAAS), was used in this study. The high resistance of Q2 to drought was confirmed in a previous study [54]. This experiment was conducted in a controlled greenhouse (20 °C, 16 h/8 h L/d, 65% humidity). Q2 seeds were sterilized with 0.1% HgCl_2_ for 10 min, and rinsed three times in the sterile water for 20 min. Seeds were put on the moist filter paper in the petri dishes for germinating at 25 °C. After 7 days, uniform seedlings were randomly selected and transplanted into plastic pots (6.5 cm × 4.5 cm), filled with a mixture of 70 g of soil, vermiculite, and sand (soil: vermiculite: sand = 2:1:1, v/v/v). The vermiculite mixture had a field capacity (FC) of 45.21% determined by the methods of Wilcox [55] and Duan et al. [56]. The plants were watered normally from germination until the three-leaf stage (at 18 days), followed by different treatments: (1) normal watering for 8 days and maintaining 75% FC (set as control, CK); (2) 8-day drought treatment (no watering), decreasing the FC to 35% (set as drought stress, DS) [57]; and (3) 7-day drought treatment followed by 1-day of rehydration (adding water), causing the FC to recover to 75% (set as re-watering, RW). This experiment sets as a completely randomized design with 3 replicates. After each treatment, the third leaf (from the top of the seedlings) was cut into five parts and mixed to form one sample for each replicate of each treatment. All samples (9 in total) were flash frozen in liquid nitrogen and stored at -80 °C prior to RNA extraction.

### Determination of physiological parameters and hormone contents

The uniform-growing seedlings were selected under the CK, DS, and RW treatments. After recording the fresh weight of the plants, seedlings were divided into shoots and roots. The content of five endogenous plant hormones (IAA, ABA, ZT, GA3, and SA) in the leaves of rapeseed seedlings under different treatments was measured via HPLC-MS/MS analysis [58, 59].

### RNA extraction and testing

The total RNA was extracted from each sample according to the manufacturer’s instruction using Trizol reagent (Invitrogen, Burlington, ON, Canada). RNA quality was assessed using several methods: firstly, the RNA degradation and contamination were monitored using the 1% agarose gel; secondly, RNA purity was detected by the Nanophotometer spectrophotometer (IMPLEN, CA, USA). Thirdly, RNA concentration was measured using the Qubit RNA Assay Kit in Qubit 2.0 Fluorometer (Life Technologies, CA, USA). Finally, RNA integrity was assessed using the RNA Nano 6000 Assay Kit of the Bioanalyzer 2100 system (Agilent Technologies, CA, USA).

### Library construction, sequencing, and mapping to the reference genome

The library preparation and deep sequencing were performed by the Novogene Bioinformatics Technology Cooperation (Beijing, China). A total amount of 3 µg RNA per sample was used as input material for the RNA sample preparations. Firstly, ribosomal RNA was removed by Epicentre Ribo-zero™ rRNA Removal Kit (Epicentre, USA), and rRNA free residue was cleaned up by ethanol precipitation. Subsequently, sequencing libraries were generated using the rRNA-depleted RNA by NEBNext^®^ Ultra™ Directional RNA Library Prep Kit for Illumina^®^ (NEB, USA) following manufacturer’s recommendations. Briefly, fragmentation was carried out using divalent cations under elevated temperature in NEBNext First Strand Synthesis Reaction Buffer (5X). First strand cDNA was synthesized using random hexamer primer and M-MuLV Reverse Transcriptase (RNaseH-). Second strand cDNA synthesis was subsequently performed using DNA Polymerase I and RNase H. In the reaction buffer, dNTPs with dTTP were replaced by dUTP. Remaining overhangs were converted into blunt ends via exonuclease/polymerase activities. After adenylation of 3’ ends of DNA fragments, NEBNext Adaptor with hairpin loop structure were ligated to prepare for hybridization. In order to select cDNA fragments of preferentially 150 ~ 200 bp in length, the library fragments were purified with AMPure XP system (Beckman Coulter, Beverly, USA). Then 3 µl USER Enzyme (NEB, USA) was used with size-selected, adaptor-ligated cDNA at 37 °C for 15 min followed by 5 min at 95 °C before PCR. Then PCR was performed with Phusion High-Fidelity DNA polymerase, Universal PCR primers and Index (X) Primer. At last, products were purified (AMPure XP system) and library quality was assessed on the Agilent Bioanalyzer 2100 system. Each sample was processed individually, and named as CKQ2-1, CKQ2-2, CKQ2-3, DSQ2-1, DSQ2-2, DSQ2-3, RWQ2-1, RWQ2-2, and RWQ2-3, resulting in a total of 9 libraries. All libraries were sequenced on the Illumina Hiseq 2500 platform, generating 125 bp paired-end reads. The original data of this experiment has been uploaded to the NCBI database, and the relevant accession numbers is PRJNA876031. Quality parameters such as Q20, Q30, and GC content of the clean data were calculated. Clean reads from each sample library were mapped to the reference database of *Brassica* genomes (https://appliedbioinformatics.com.au/gb2/gbrowse/BnapusPan/) using Bowtie v2.0.6.

### Identification of lncRNAs

Before screening, the Cuffmerge software was used to merge transcripts spliced from each sample and remove transcripts with an uncertain chain direction to obtain complete transcriptome information for this sequencing. Then, we screened the combined transcript set for lncRNA using the following steps: (1) Select transcripts with a number of exons ≥ 2; (2) Select transcripts with a length > 200 bp; (3) Screen out transcripts that overlap the exon region of the database annotation using Cuffcompare software and include lncRNA that overlap the exon region of the transcript splicing in the database as database annotation lncRNA for subsequent analysis; (4) Calculate the expression amount of each transcript using Cuffquant, and select transcripts with FPKM ≥ 0.5; (5) For spliced transcripts, we employed bioinformatics tools such as CPAT, CNCI, PfamScan, and phyloCSF to comprehensively assess the coding potential of transcripts for screening, taking the intersection of predicted transcripts without coding potential in the analyzed software results. Subsequently, we identified transcripts that were predicted to have coding potential by at least one of the coding potential prediction software as TUCP (transcripts of uncertain coding potential) for this analysis. Among these transcripts, there may be a subset of lncRNA with certain coding potential, and therefore, we included them as a separate category of transcripts for subsequent analysis [60].

### Quantification of gene expression level, and gene expression profiles

Firstly, the Cuffdiff software was used to calculate the FPKMs (fragments per kilo-base of exon per million fragments mapped) of both lncRNAs and mRNAs in each sample. Then, the FPKM method was used to calculate the expression level of each transcript. Finally, the average value of the three replicates from the same treatment was recorded as the expression intensity of specific genes [61, 62]. Differentially expressed lncRNAs and mRNAs were identified using the Cufflinks software with a less stringent threshold of *q*-value ≤ 0.05 for the significant gene expression comparison between samples. The *q*-value, which is the corrected *p*-value, was used to estimate the false discovery rate (FDR) in multiple comparisons [63]. The expressions of lncRNAs and mRNAs were then compared under different treatments (DS/CK, RW/DS).

### Construction of the lncRNA-mRNA co-expression network

Since the function of lncRNAs is not well-defined, a commonly used method to predict their functional mechanism is based on co-expressed mRNAs [64]. Moreover, previous studies have shown that some lncRNAs can form “lncRNA-mRNA pairs” with nearby protein-coding genes, which can affect their function [65]. To explore the co-expression relationship between lncRNAs and mRNAs, we constructed a co-expression network using the method described by Wang et al. [66]. Pearson’s correlation coefficient (PCC) and *p*-value were calculated for each lncRNA-mRNA pair, and we selected pairs with |PCC value| ≥ 0.95 and *p* < 0.05 to construct the network. The network was visualized using Cytoscape (v3.7.1; https://cytoscape.org/).

### GO and KEGG enrichment analysis

We performed GO enrichment analysis on the target genes that were co-expressed with differentially expressed lncRNAs using the GOseq R package [67]. GO terms were used to classify the genes into different categories, such as biological processes, molecular functions, and cellular components. The threshold of *p*-value < 0.05 was used to determine significantly enriched GO terms.

The KOBAS software was used to detect the statistical enrichment of differentially expressed lncRNAs target genes in the KEGG pathway (http://www.genome.ad.jp/kegg/) [68]. We considered a *p*-value < 0.05 to indicate statistically significant differences between samples.

### Validation of DE-lncRNAs through real-time quantitative PCR

To obtain the first-strand cDNA, the total RNA of each sample was treated with RNase-free DNase, and then the RevertAid First-Strand cDNA Synthesis Kit (Fermentas, USA) was used. For real-time PCR, a 10 µl reaction system was prepared on the ABI 700 Real-time PCR platform containing approximately 0.5 ng of cDNA, 2.5 µl of 1.2 µM mixture of forward and reverse primers, and 5 µl of master mix, as directed by the SYBR Green PCR Master Mix system (Takara Co. Ltd., Japan). The PCR amplification conditions were set as follows: one cycle of 95 °C for 30 s, followed by 40 cycles of 95 °C for 5 s, and 60 °C for 30 s. We used Primer Premier 5 software to design and analyze PCR primers for the detection of candidate lncRNAs, which were listed in Supplementary Table 8. In our experiment, random primers were used for reverse transcription. For each experiment, three independent biological replicates were performed, and for each sample in the RT-qPCR reaction, three biological and three technical replicates were performed.

## Conclusion

In this study, seedlings of a drought-tolerant rapeseed germplasm were subjected to drought and rehydration treatments. The transcriptomes of lncRNAs and mRNAs were analyzed through sequencing of the leaves under CK, DS, and RW conditions. A total of 184 DE-lncRNAs were found to be consistently expressed in the two comparison groups of DS vs. CK and RW vs. DS. A lncRNA-mRNA network was established to understand the role of lncRNAs in responding to drought stress and rehydration. The results showed that the plant hormone signal transduction pathway was the most significantly enriched in both DS vs. CK and RW vs. DS according to the transcriptome assay. Several enriched candidate mRNAs affecting phytohormone function were identified, and they played a role in drought stress tolerance in rapeseed seedlings through interaction with related lncRNAs. The study revealed a multiple plant hormone signaling mediated network regulating drought resistance in rapeseed through the lncRNA-mRNA network, with lncRNAs induced by plant hormone signals involved in ABA, IAA, CTKs, and GAs signal transduction pathways. This is the first discovery of the lncRNA-mRNA network involved in rapeseed under drought stress and drought-rehydration process. The findings provided new insights into lncRNAs in response to water loss & supply, enriched the related plant hormone signal transduction pathways and key genes for understanding drought tolerance mechanisms in plants. Manipulation of the candidate lncRNAs or the co-expressing mRNAs may enhance crop drought tolerance in the future.

### Electronic supplementary material

Below is the link to the electronic supplementary material.

Supplementary Material 1

Supplementary Material 2

Supplementary Material 3

Supplementary Material 4

Supplementary Material 5

Supplementary Material 6

Supplementary Material 7

Supplementary Material 8

Supplementary Material 9

## Data Availability

The original data of this experiment has been uploaded to the NCBI database (https://www.ncbi.nlm.nih.gov/bioproject/PRJNA876031/), and the relevant accession numbers is PRJNA876031.

## References

[1] Ault TR. On the essentials of Drought in a changing climate. Science. 2020;368:256–60. 10.1126/science.aaz5492. 32299944 10.1126/science.aaz5492

[2] Shinozaki K, Yamaguchi-Shinozaki K. Gene networks involved in Drought stress response and tolerance. J Exp Bot. 2007;58:221–7. 10.1093/jxb/erl164. 17075077 10.1093/jxb/erl164

[3] Gui Y, Sheteiwy M, Shuangguo Z, Zhu L, Batool A, Jia T, Xiong Y. Differentiate responses of tetraploid and Hexaploid Wheat (*Triticum Aestivum* L.) to moderate and severe Drought stress: a cue of wheat domestication. Plant Signal Behav. 2020;16:1839710. 10.1080/15592324.2020.1839710. 33126814 10.1080/15592324.2020.1839710 PMC7781840

[4] Yoshida T, Mogami J, Yamaguchi-Shinozaki K. ABA-Dependent and ABA-Independent signaling in response to osmotic stress in plants. Curr Opin Plant Biol. 2014;21:133–9. 10.1016/j.pbi.2014.07.009. 25104049 10.1016/j.pbi.2014.07.009

[5] Singh D, Laxmi A. Transcriptional regulation of Drought Response: a Tortuous Network of Transcriptional factors. Front Plant Sci. 2015;6:895. 10.3389/fpls.2015.00895. 26579147 10.3389/fpls.2015.00895 PMC4625044

[6] Cai S, Guang C, Wang Y, Huang Y, Marchant D, Wang Y, Yang Q, Dai F, Hills A, Franks P, et al. Evolutionary conservation of ABA signaling for Stomatal Closure. Plant Physiol. 2017;174:732–47. 10.1104/pp.16.01848. 28232585 10.1104/pp.16.01848 PMC5462018

[7] Huang GT, Ma SL, Bai LP, Zhang L, Ma H, Jia P, Liu J, Zhong M, Guo ZF. Signal Transduction during Cold, Salt, and Drought stresses in plants. Mol Biol Rep. 2011;39:969–87. 10.1007/s11033-011-0823-1. 21573796 10.1007/s11033-011-0823-1

[8] Unver T, Tombuloglu H. Barley long non-coding RNAs (lncRNA) responsive to excess boron. Genomics. 2020;112:1947–55. 10.1016/j.ygeno.2019.11.007. 31730798 10.1016/j.ygeno.2019.11.007

[9] Yu Y, Zhou YF, Feng YZ, He H, Lian JP, Yang YW, Lei MQ, Zhang YC, Chen YQ. Transcriptional Landscape of Pathogen-Responsive LncRNAs in Rice unveils the role of ALEX1 in Jasmonate Pathway and Disease Resistance. Plant Biotechnol J. 2020;18:679–90. 10.1111/pbi.13234. 31419052 10.1111/pbi.13234 PMC7004900

[10] Chen K, Huang Y, Liu C, Liang Y, Li M. Transcriptome Profile Analysis of Arabidopsis reveals the Drought stress-induced long non-coding RNAs Associated with Photosynthesis, Chlorophyll synthesis, fatty acid synthesis and degradation. Front Plant Sci. 2021;12:643182. 10.3389/fpls.2021.643182. 34113361 10.3389/fpls.2021.643182 PMC8185149

[11] Qin T, Zhao H, Cui P, Albesher N, Xiong LA, Nucleus-Localized. Long non-coding RNA enhances Drought and Salt stress tolerance. Plant Physiol. 2017;175:1321–36. 10.1104/pp.17.00574. 28887353 10.1104/pp.17.00574 PMC5664461

[12] Yang X, Liu C, Niu X, Wang L, Li L, Yuan Q, Pei X. Research on LncRNA related to Drought Resistance of Shanlan Upland Rice. BMC Genomics. 2022;23:336. 10.1186/s12864-022-08546-0. 35490237 10.1186/s12864-022-08546-0 PMC9055766

[13] Lu X, Chen X, Mu M, Wang J, Wang X, Wang D, Yin Z, Fan W, Wang S, Guo L, et al. Genome-wide analysis of long noncoding RNAs and their responses to Drought stress in cotton (*Gossypium Hirsutum* L). PLoS ONE. 2016;11:e0156723. 10.1371/journal.pone.0156723. 27294517 10.1371/journal.pone.0156723 PMC4905672

[14] Ding Z, Tie W, Fu L, Yan Y, Liu G, Yan W, Li Y, Wu C, Zhang J, Hu W, Strand-Specific. RNA-Seq based Identification and Functional Prediction of Drought-Responsive LncRNAs in Cassava. BMC Genomics. 2019;20:214. 10.1186/s12864-019-5585-5. 30866814 10.1186/s12864-019-5585-5 PMC6417064

[15] Xiao L, Shang XH, Cao S, Xie XY, Zeng WD, Lu LY, Chen SB, Yan HB. Comparative physiology and transcriptome analysis allows for identification of LncRNAs Imparting Tolerance to Drought stress in Autotetraploid Cassava. BMC Genomics. 2019;20:514. 10.1186/s12864-019-5895-7. 31226927 10.1186/s12864-019-5895-7 PMC6588902

[16] Eom SH, Lee HJ, Lee JH, Wi SH, Kim SK, Hyun TK. Identification and Functional Prediction of Drought-Responsive Long non-coding RNA in Tomato. Agronomy. 2019;9. 10.3390/agronomy9100629.

[17] Qiu CW, Zhao J, Chen Q, Wu F. Genome-wide characterization of Drought stress responsive long non-coding RNAs in tibetan wild barley. Environ Exp Bot. 2019;164. 10.1016/j.envexpbot.2019.05.002.

[18] Zhu M, Monroe JG, Suhail Y, Villiers F, Mullen J, Pater D, Hauser F, Jeon BW, Bader JS, Kwak JM, et al. Molecular and systems approaches towards Drought-Tolerant Canola crops. New Phytol. 2016;210:1169–89. 10.1111/nph.13866. 26879345 10.1111/nph.13866

[19] Tan X, Li S, Hu L, Zhang C. Genome-wide analysis of long non-coding RNAs (LncRNAs) in two contrasting rapeseed (*Brassica Napus* L.) genotypes subjected to Drought stress and re-watering. BMC Plant Biol. 2020;20:81. 10.1186/s12870-020-2286-9. 32075594 10.1186/s12870-020-2286-9 PMC7032001

[20] Ullah A, Ullah A, Manghwar H, Shaban M, Khan AH, Akbar A, Ali U, Ali E, Fahad S. Phytohormones enhanced Drought Tolerance in plants: a coping strategy. Environ Sci Pollut R Int. 2018;25:33103–18. 10.1007/s11356-018-3364-5. 10.1007/s11356-018-3364-5 30284160

[21] Chhaya; Yadav B, Jogawat A, Gnanasekaran P, Kumari P, Lakra N, Lal SK, Pawar J, Narayan OP. An overview of recent Advancement in Phytohormones-Mediated Stress Management and Drought Tolerance in Crop plants. Plant Gene. 2021;25:100264. 10.1016/j.plgene.2020.100264. 10.1016/j.plgene.2020.100264

[22] Tan S, Alex R, Unver T, Editorial. Transcriptional and post-transcriptional regulations in agricultural species after stresses. Front Genet. 2023;13:1127832. 10.3389/fgene.2022.1127832. 36685856 10.3389/fgene.2022.1127832 PMC9846490

[23] Bakir Y, Eldem V, Zararsiz G, Unver T. Global transcriptome analysis reveals differences in Gene expression patterns between nonhyperhydric and hyperhydric Peach leaves. Plant Genome. 2016;9. 10.3835/plantgenome2015.09.0080. 10.3835/plantgenome2015.09.0080 27898837

[24] Xu Z, Zhou G, Shimizu H. Plant responses to Drought and Rewatering. Plant Signal Behav. 2010;5:649–54. 10.4161/psb.5.6.11398. 20404516 10.4161/psb.5.6.11398 PMC3001553

[25] Mehrotra R, Bhalothia P, Bansal P, Basantani MK, Bharti V, Mehrotra S. Abscisic acid and abiotic stress tolerance-different tiers of regulation. J Plant Physiol. 2014;171:486–96. 10.1016/J.JPLPH.2013.12.007. 24655384 10.1016/J.JPLPH.2013.12.007

[26] Davies W, Kudoyarova G, Hartung W. Long-distance ABA signaling and its relation to other signaling pathways in the detection of Soil Drying and the mediation of the Plant’s response to Drought. J Plant Growth Regul. 2005;24:285–95. 10.1007/s00344-005-0103-1. 10.1007/s00344-005-0103-1

[27] Sheteiwy MS, Abd Elgawad H, Xiong YC, Macovei A, Brestic M, Skalicky M, Shaghaleh H, Alhaj Hamoud Y, El-Sawah AM. Inoculation with Bacillus Amyloliquefaciens and Mycorrhiza confers tolerance to Drought stress and improve seed yield and quality of soybean plant. Physiol Plant. 2021;172:2153–69. 10.1111/ppl.13454. 33964177 10.1111/ppl.13454

[28] Melcher K, Ng LM, Zhou XE, Soon FF, Xu Y, Suino-Powell KM, Park SY, Weiner JJ, Fujii H, Chinnusamy V, et al. A gate-latch-lock mechanism for hormone signalling by Abscisic Acid receptors. Nature. 2009;462:602–8. 10.1038/nature08613. 19898420 10.1038/nature08613 PMC2810868

[29] Eren H, Pekmezci MY, Okay S, Turktas M, Inal B, Ilhan E, Atak M, Erayman M, Unver T. Hexaploid wheat (Triticum aestivum) root miRNome analysis in response to salt stress. Ann Appl Biol. 2015;167:208–16. 10.1111/aab.12219. 10.1111/aab.12219

[30] Merlot S, Gosti F, Guerrier D, Vavasseur A, Giraudat J. The ABI1 and ABI2 protein phosphatases 2 C Act in a negative Feedback Regulatory Loop of the Abscisic Acid Signalling Pathway. Plant J. 2001;25:295–303. 10.1046/j.1365-313x.2001.00965.x. 11208021 10.1046/j.1365-313x.2001.00965.x

[31] Zhang F, Fu X, Lv Z, Shen Q, Yan T, Jiang W, Wang G, Sun X, Tang K. Type 2 C Phosphatase 1 of Artemisia Annua L. Is a Negative Regulator of ABA Signaling. Biomed Res Int. 2014;2014(521794). 10.1155/2014/521794. 10.1155/2014/521794 PMC4228716 25530962

[32] Xiang Y, Sun X, Gao S, Qin F, Dai M. Deletion of an endoplasmic reticulum stress response element in a ZmPP2C-A gene facilitates Drought Tolerance of Maize Seedlings. Mol Plant. 2017;10:456–69. 10.1016/j.molp.2016.10.003. 27746300 10.1016/j.molp.2016.10.003

[33] Zhang F, Wei Q, Shi J, Jin X, He Y, Zhang Y, Luo Q, Wang Y, Chang J, Yang GX, et al. Brachypodium Distachyon BdPP2CA6 interacts with BdPYLs and BdSnRK2 and positively regulates Salt Tolerance in Transgenic *Arabidopsis*. Front Plant Sci. 2017;8:64. 10.3389/fpls.2017.00264. 28293246 10.3389/fpls.2017.00264 PMC5329023

[34] Min DH, Xue FY, Ma Y, Chen M, Xu ZS, Li LC, Diao XM, Jia GQ, Ma YZ. Characteristics of PP2C Gene Family in Foxtail Millet (*Setaria Italica*). Acta Agron Sinica. 2013;39:2135. 10.3724/SP.J.1006.2013.02135. 10.3724/SP.J.1006.2013.02135

[35] De Smet I, Jürgens G. Patterning the Axis in Plants-Auxin in control. Curr Opin Genet Dev. 2007;17:337–43. 10.1016/j.gde.2007.04.012. 17627808 10.1016/j.gde.2007.04.012

[36] Zhao Y, Wu L, Fu Q, Wang D, Li J, Yao B, Yu S, Jiang L, Qian J, Zhou X, et al. INDITTO2 transposon conveys auxin-mediated DRO1 transcription for Rice Drought Avoidance. Plant Cell Environ. 2021;44:1846–57. 10.1111/pce.14029. 33576018 10.1111/pce.14029

[37] Chapman EJ, Estelle M. Mechanism of Auxin-regulated gene expression in plants. Annu Rev Genet. 2009;43:265–85. 10.1146/annurev-genet-102108-134148. 19686081 10.1146/annurev-genet-102108-134148

[38] Luo J, Zhou JJ, Zhang JZ, Aux. /IAA Gene Family in plants: molecular structure, regulation, and function. Int J Mol Sci. 2018;19:259. 10.3390/ijms19010259. 29337875 10.3390/ijms19010259 PMC5796205

[39] Mano Y, Nemoto K. The pathway of Auxin Biosynthesis in plants. J Exp Bot. 2012;638:2853–72. 10.1093/jxb/ers091. 10.1093/jxb/ers091 22447967

[40] Mellor N, Bennett MJ, King JR. GH3-Mediated Auxin Conjugation can result in either transient or oscillatory transcriptional auxin responses. Bull Math Biol. 2016;78:210–34. 10.1007/s11538-015-0137-x. 26767838 10.1007/s11538-015-0137-x

[41] Kant S, Bi YM, Zhu T, Rothstein S. SAUR39, a small Auxin-Up RNA gene, acts as a negative Regulator of Auxin Synthesis and Transport in Rice. Plant Physiol. 2009;151:691–701. 10.1104/pp.109.143875. 19700562 10.1104/pp.109.143875 PMC2754634

[42] Hai NN, Chuong NN, Tu NH, Kisiala A, Hoang XL, Thao NP. Role and regulation of cytokinins in Plant Response to Drought stress. Plants. 2020;9:422. 10.3390/plants9040422. 32244272 10.3390/plants9040422 PMC7238249

[43] Iqbal S, Wang X, Mubeen I, Kamran M, Kanwal I, Díaz G, Abbas A, Parveen A, Atiq M, Alshaya H, et al. Phytohormones trigger Drought Tolerance in Crop plants: Outlook and Future perspectives. Front Plant Sci. 2022;12:799318. 10.3389/fpls.2021.799318. 35095971 10.3389/fpls.2021.799318 PMC8792739

[44] Kang NY, Cho C, Kim NY, Kim J. Cytokinin receptor-dependent and receptor-independent pathways in the Dehydration response of *Arabidopsis* Thaliana. J Plant Physiol. 2012;169:1382–91. 10.1016/j.jplph.2012.05.007. 22704545 10.1016/j.jplph.2012.05.007

[45] Tran LSP, Urao T, Qin F, Maruyama K, Kakimoto T, Shinozaki K, Yamaguchi-Shinozaki K. Functional analysis of AHK1/ATHK1 and cytokinin receptor histidine kinases in response to Abscisic Acid, Drought, and salt stress in Arabidopsis. Proc Natl Acad Sci U S A. 2007;104:20623–8. 10.1073/pnas.0706547105. 18077346 10.1073/pnas.0706547105 PMC2154481

[46] Nishiyama R, Watanabe Y, Leyva-González M, Ha C, Fujita Y, Tanaka M, Seki M, Yamaguchi-Shinozaki K, Shinozaki K, Herrera-Estrella L et al. *Arabidopsis* AHP2, AHP3, and AHP5 Histidine Phosphotransfer Proteins Function as Redundant Negative Regulators of Drought Stress Response. *Proc. Natl. Acad. Sci* 2013, *110*, 4840–4845. 10.1073/pnas.1302265110. 10.1073/pnas.1302265110 PMC3606972 23487796

[47] Yokoyama A, Yamashino T, Amano YI, Tajima Y, Imamura A, Sakakibara H, Mizuno T, Type. -B ARR Transcription Factors, ARR10 and ARR12, are implicated in cytokinin-mediated regulation of Protoxylem differentiation in roots of *Arabidopsis* Thaliana. Plant cell Physiol. 2007;48:84–96. 10.1093/pcp/pcl040. 17132632 10.1093/pcp/pcl040

[48] Pimenta Lange MJ, Lange T. Gibberellin Biosynthesis and the regulation of Plant Development. Plant Biol (Stuttg). 2006;8:281–90. 10.1055/s-2006-923882. 16807819 10.1055/s-2006-923882

[49] Vishal B, Kumar P. Regulation of seed germination and Abiotic stresses by Gibberellins and Abscisic Acid. Front Plant Sci. 2018;9:838. 10.3389/fpls.2018.00838. 29973944 10.3389/fpls.2018.00838 PMC6019495

[50] Shohat H, Cheriker H, Kilambi HV, Illouz Eliaz N, Blum S, Amsellem Z, Tarkowská D, Aharoni A, Eshed Y, Weiss D. Inhibition of Gibberellin Accumulation by Water Deficiency promotes fast and long-term ‘Drought Avoidance’ responses in Tomato. New Phytol. 2021;232:1985–98. 10.1111/nph.17709. 34541677 10.1111/nph.17709

[51] Shohat H, Eliaz NI, Weiss D. Gibberellin in Tomato: metabolism, Signaling and Role in Drought responses. Mol Hortic. 2021;1:15. 10.1186/s43897-021-00019-4. 37789477 10.1186/s43897-021-00019-4 PMC10515025

[52] Hirano K, Ueguchi-Tanaka M, Matsuoka M. GID1-Mediated Gibberellin signaling in plants. Trends Plant Sci. 2008;13:192–9. 10.1016/j.tplants.2008.02.005. 18337155 10.1016/j.tplants.2008.02.005

[53] Ueguchi-Tanaka M, Nakajima M, Katoh E, Ohmiya H, Asano K, Saji S, Hongyu X, Ashikari M, Kitano H, Yamaguchi I, et al. Molecular interactions of a Soluble Gibberellin receptor, GID1, with a Rice DELLA protein, SLR1, and Gibberellin. Plant Cell. 2007;19:2140–55. 10.1105/tpc.106.043729. 17644730 10.1105/tpc.106.043729 PMC1955699

[54] Xiao QS. Drought-related Gene Expression Analysis during Drought Stress in Rapeseed (*Brassica napus* L.). Master’s Thesis, Oil Crops Research Institute Chinese Academy of Agricultural Sciences, Wuhan, 2011.

[55] Wilcox JC. Time of sampling after an irrigation to Determine Field Capacity of Soil. Can J Soil Sci. 1965;45:171–6. 10.4141/cjss65-024. 10.4141/cjss65-024

[56] Duan X, Xie Y, Liu G, Gao X, Lu H. Field Capacity in Black Soil Region, Northeast China. Chin Geogr Sci. 2010;20:406–13. 10.1007/s11769-010-0414-4. 10.1007/s11769-010-0414-4

[57] Naeem MS, Dai L, Ahmad F, Ahmad A, Li J, Zhang C. AM1 is a potential ABA substitute for Drought Tolerance as revealed by physiological and ultra-structural responses of Oilseed rape. Acta Physiol Plant. 2016;38:183. 10.1007/s11738-016-2190-y. 10.1007/s11738-016-2190-y

[58] Li Y, Zhou C, Yan X, Zhang J, Xu J. Simultaneous analysis of ten phytohormones in Sargassum Horneri by High-Performance Liquid Chromatography with Electrospray Ionization Tandem Mass Spectrometry. J Sep Sci. 2016;39:1804–13. 10.1002/jssc.201501239. 26990813 10.1002/jssc.201501239

[59] Floková K, Tarkowská D, Miersch O, Strnad M, Wasternack C, Novák O. UHPLC–MS/MS Based Target profiling of stress-Induced Phytohormones. Phytochemistry. 2014;105:147–57. 10.1016/j.phytochem.2014.05.015. 24947339 10.1016/j.phytochem.2014.05.015

[60] Cabili MN, Trapnell C, Goff LA, Koziol MJ, Tazón-Vega B, Regev A, Rinn JL. Integrative annotation of human large intergenic noncoding RNAs reveals global properties and specific subclasses. Genes Dev. 2011;25:1915–27. 10.1101/gad.17446611. 21890647 10.1101/gad.17446611 PMC3185964

[61] Trapnell C, Williams BA, Pertea G, Mortazavi A, Kwan G, van Baren MJ, Salzberg SL, Wold BJ, Pachter L. Transcript Assembly and quantification by RNA-Seq reveals unannotated transcripts and isoform switching during cell differentiation. Nat Biotechnol. 2010;28:511–5. 10.1038/nbt.1621. 20436464 10.1038/nbt.1621 PMC3146043

[62] Trapnell C, Roberts A, Goff L, Pertea G, Kim D, Kelley DR, Pimentel H, Salzberg SL, Rinn JL, Pachter L. Differential Gene and transcript expression analysis of RNA-Seq experiments with TopHat and Cufflinks. Nat Protoc. 2012;7:562–78. 10.1038/nprot.2012.016. 22383036 10.1038/nprot.2012.016 PMC3334321

[63] Lai Y. A statistical method for the Conservative Adjustment of false Discovery rate (q-Value). BMC Bioinformatics. 2017;18:69. 10.1186/s12859-017-1474-6. 28361675 10.1186/s12859-017-1474-6 PMC5374657

[64] Liao Q, Liu C, Yuan X, Kang S, Miao R, Xiao H, Zhao G, Luo H, Bu D, Zhao H, et al. Large-scale prediction of long non-coding RNA functions in a coding-non-coding gene Co-expression Network. Nucleic Acids Res. 2011;39:3864–78. 10.1093/nar/gkq1348. 21247874 10.1093/nar/gkq1348 PMC3089475

[65] Pan F, Yao J, Chen Y, Zhou C, Geng P, Mao H, Fang XA. Novel long non-coding RNA FOXCUT and MRNA FOXCUT Pair promote progression and predict poor prognosis in esophageal squamous cell carcinoma. Int J Clin Exp Patho. 2014;7:2838–49. PMC4097289 25031703

[66] Wang R, Zou J, Meng J, Wang J. Integrative Analysis of Genome-Wide LncRNA and MRNA expression in newly synthesized Brassica Hexaploids. Ecol Evol. 2018;8:6034–52. 10.1002/ece3.4152. 29988444 10.1002/ece3.4152 PMC6024132

[67] Young MD, Wakefield MJ, Smyth GK, Oshlack A. Gene Ontology Analysis for RNA-Seq: accounting for Selection Bias. Genome Biol. 2010;11:R14. 10.1186/gb-2010-11-2-r14. 20132535 10.1186/gb-2010-11-2-r14 PMC2872874

[68] Mao X, Cai T, Olyarchuk JG, Wei L. Automated Genome Annotation and Pathway Identification using the KEGG Orthology (KO) as a controlled vocabulary. Bioinformatics. 2005;21:3787–93. 10.1093/bioinformatics/bti430. 15817693 10.1093/bioinformatics/bti430

