# Supplementary material for: Transcriptome analysis suggested that lncRNAs regulate rapeseed seedlings in responding to drought stress by coordinating the phytohormone signal transduction pathways
Source: BMC Genomics. 2024 Jul 19;25:704. doi: 10.1186/s12864-024-10624-4 (PMC11264961; doi:10.1186/s12864-024-10624-4)
Supplement: Supplementary file 1 — Supplementary Material 1 [file 12864_2024_10624_MOESM1_ESM.pdf]

Gene Ontology (GO) classifications of the target genes of DE-lncRNAs which co-expressed in two comparisons

Category	Items Number of Genes	Number of Genes
Biological process	oxidation-reduction process	78
	protein dephosphorylation	12
	dephosphorylation	12
	response to abiotic stimulus	5
	embryo development	5
	single-organism developmental process	5
	anatomical structure development	5
	coenzyme M biosynthetic process	2
	coenzyme M metabolic process	2
Cellular component	CCAAT-binding factor complex	7
Molecular function	nucleic acid binding transcription factor activity	52
	sequence-specific DNA binding transcription factor activity	52
	sequence-specific DNA binding	34
	phosphoprotein phosphatase activity	12
	protein serine/threonine phosphatase activity	12
	amine-lyase activity	6
	strictosidine synthase activity	6
	urate oxidase activity	2
	oxidoreductase activity, acting on other nitrogenous compounds as donors	2
	oxidoreductase activity, acting on other nitrogenous compounds as donors, oxygen as acceptor	2
