# Supplementary material for: Transcriptome analysis suggested that lncRNAs regulate rapeseed seedlings in responding to drought stress by coordinating the phytohormone signal transduction pathways
Source: BMC Genomics. 2024 Jul 19;25:704. doi: 10.1186/s12864-024-10624-4 (PMC11264961; doi:10.1186/s12864-024-10624-4)
Supplement: Supplementary file 3 — Supplementary Material 3 [file 12864_2024_10624_MOESM3_ESM.pdf]

List of significant pathways identified by Kyoto Encyclopedia of Genes and Genomes (KEGG) enrichment analysis of the target genes of DE-lncRNA of significant pathways identified by KEGG enrichment analysis of the target genes of DE-lncRNAs which co-expressed in two comparisons

KEGG ID	Pathway	Number of Transcripts
ko04075	Plant hormone signal transduction	33
ko01200	Carbon metabolism	21
ko00010	Glycolysis / Gluconeogenesis	13
ko00250	Alanine, aspartate and glutamate metabolism	11
ko00052	Galactose metabolism	10
ko00071	Fatty acid degradation	9
ko00280	Valine, leucine and isoleucine degradation	9
ko00310	Lysine degradation	9
ko00630	Glyoxylate and dicarboxylate metabolism	9
ko00350	Tyrosine metabolism	8
ko00592	alpha-Linolenic acid metabolism	8
ko00330	Arginine and proline metabolism	7
ko00380	Tryptophan metabolism	7
ko00910	Nitrogen metabolism	6
ko00410	beta-Alanine metabolism	5
ko01040	Biosynthesis of unsaturated fatty acids	5
ko00300	Lysine biosynthesis	4
ko00640	Propanoate metabolism	2
