# Supplementary material for: Transcriptome analysis suggested that lncRNAs regulate rapeseed seedlings in responding to drought stress by coordinating the phytohormone signal transduction pathways
Source: BMC Genomics. 2024 Jul 19;25:704. doi: 10.1186/s12864-024-10624-4 (PMC11264961; doi:10.1186/s12864-024-10624-4)
Supplement: Supplementary file 4 — Supplementary Material 4 [file 12864_2024_10624_MOESM4_ESM.pdf]

Relative expression data measured by qRT-PCRs and RNA-seq

Gene name	Sample name	Relative expression measured by RT-qPCR	Relative expression measured by RNA-seq
XLOC_000799	DS vs. CK	-1.40586	-2.54466
XLOC_074921	DS vs. CK	-3.56702	-2.33727
XLOC_001194	DS vs. CK	-0.72385	-1.48253
XLOC_032712	DS vs. CK	-2.15738	-3.08685
XLOC_008374	DS vs. CK	-2.28365	-3.30137
XLOC_083471	DS vs. CK	1.27163	0.64061
XLOC_087430	DS vs. CK	2.31351	1.50449
XLOC_095219	DS vs. CK	2.62942	1.96666
XLOC_071711	DS vs. CK	3.35975	2.46565
XLOC_071682	DS vs. CK	0.88325	1.10827
XLOC_000799	RW vs. DS	1.86406	2.21598
XLOC_074921	RW vs. DS	1.95731	1.44761
XLOC_001194	RW vs. DS	2.68592	3.62206
XLOC_032712	RW vs. DS	3.32137	2.02107
XLOC_008374	RW vs. DS	2.81716	3.83526
XLOC_083471	RW vs. DS	-1.09645	-2.35881
XLOC_087430	RW vs. DS	-3.18716	-4.24863
XLOC_095219	RW vs. DS	-1.98647	-3.26534
XLOC_071711	RW vs. DS	-2.84524	-3.61969
XLOC_071682	RW vs. DS	-1.88673	-2.74141
