# Supplementary material for: Transcriptome analysis suggested that lncRNAs regulate rapeseed seedlings in responding to drought stress by coordinating the phytohormone signal transduction pathways
Source: BMC Genomics. 2024 Jul 19;25:704. doi: 10.1186/s12864-024-10624-4 (PMC11264961; doi:10.1186/s12864-024-10624-4)
Supplement: Supplementary file 5 — Supplementary Material 5 [file 12864_2024_10624_MOESM5_ESM.pdf]

RNA amount obtained from each treatment for RNA-seq analysis

Sample name	Concentration (ng μL^{-1})	Volume (μL)	Amount (μg)
Q2CK_1	426	32	13.632
Q2CK_2	280	32	8.96
Q2CK_3	582	32	18.624
Q2DS_1	660	32	21.12
Q2DS_2	844	32	27.008
Q2DS_3	1086	32	34.752
Q2RW_1	996	32	31.872
Q2RW_2	762	32	24.384
Q2RW_3	1250	32	40
