# Supplementary material for: Transcriptome analysis suggested that lncRNAs regulate rapeseed seedlings in responding to drought stress by coordinating the phytohormone signal transduction pathways
Source: BMC Genomics. 2024 Jul 19;25:704. doi: 10.1186/s12864-024-10624-4 (PMC11264961; doi:10.1186/s12864-024-10624-4)
Supplement: Supplementary file 6 — Supplementary Material 6 [file 12864_2024_10624_MOESM6_ESM.pdf]

Supplementary Figures

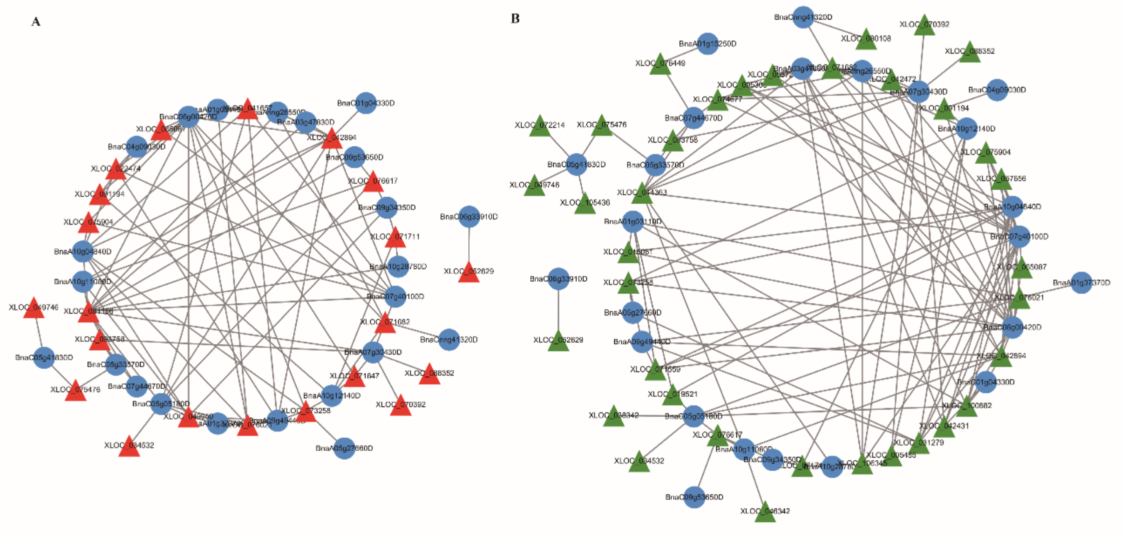

Supplementary Figure S1 LncRNA-mRNA-network analysis of the ABA signaling pathway. The circle and rectangle nodes represent lncRNAs and protein-coding genes, respectively. The up-regulated and down-regulated nodes are separately colored in red and green. Edges show regulatory interactions among nodes. (A) 21 DE-lncRNAs interact with 23 mRNAs to participate in the ABA signaling pathway in DS vs. CK. (B) 36 DE-lncRNAs interacted with 24 mRNAs to participate in the ABA signaling pathway in RW vs. DS.

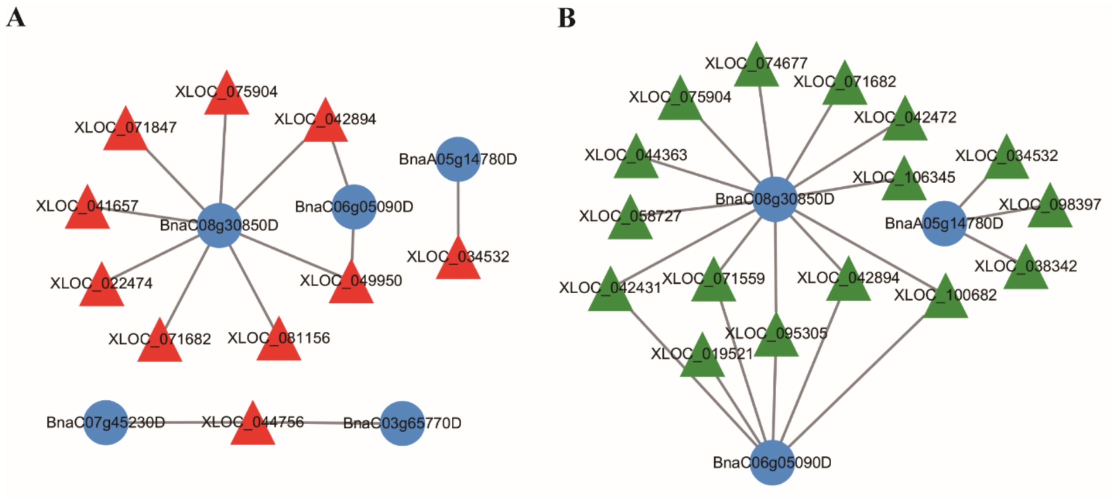

Supplementary Figure S2 LncRNA-mRNA-network analysis of the IAA signaling pathway. The circle and rectangle nodes represent lncRNAs and protein-coding genes, respectively. The up-regulated and down-regulated nodes are separately colored in red and green. Edges show regulatory interactions among nodes. (A) 10 DE-lncRNAs interact with 5 mRNAs to participate in the IAA signaling pathway in DS vs. CK. (B) 16 DE-lncRNAs interacted with 3 mRNAs to participate in the IAA signaling pathway in RW vs. DS.

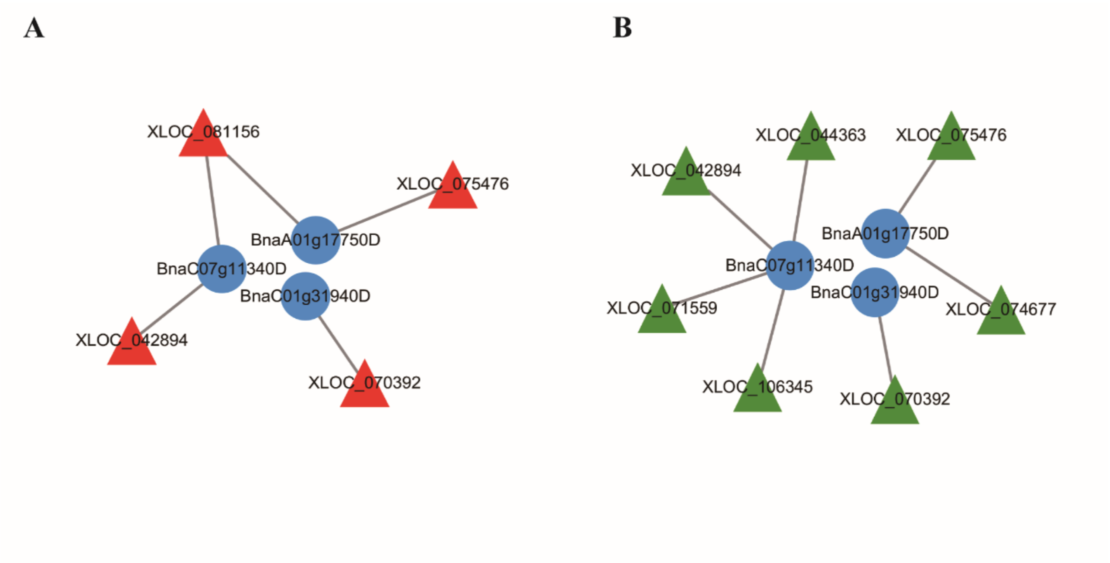

Supplementary Figure S3 LncRNA-mRNA-network analysis of the CTKs signaling pathway. The circle and rectangle nodes represent lncRNAs and protein-coding genes, respectively. The up-regulated and down-regulated nodes are separately colored in red and green. Edges show regulatory interactions among nodes. (A) 4 DE-lncRNAs interact with 3 mRNAs to participate in the CTKs signaling pathway in DS vs. CK. (B) 7 DE-lncRNAs interacted with 3 mRNAs to participate in the CTKs signaling pathway in RW vs. DS.

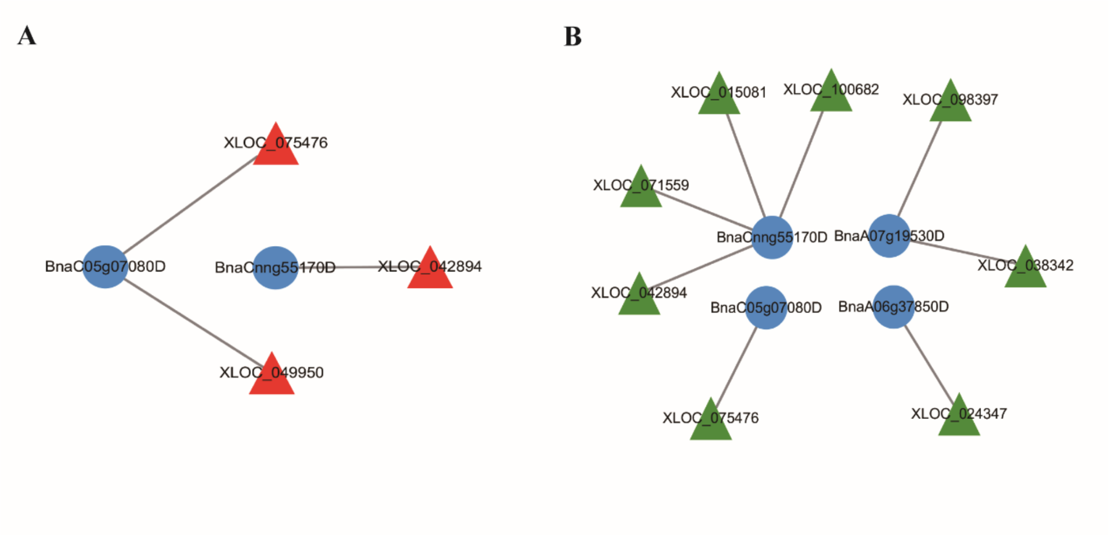

Supplementary Figure S4 LncRNA-mRNA-network analysis of the GAs signaling pathway. The circle and rectangle nodes represent lncRNAs and protein-coding genes, respectively. The up-regulated and down-regulated nodes are separately colored in red and green. Edges show regulatory interactions among nodes. (A) 3 DE-lncRNAs interact with 2 mRNAs to participate in the GAs signaling pathway in DS vs. CK. (B) 8 DE-lncRNAs interacted with 4 mRNAs to participate in the GA signaling pathway in RW vs. DS.
