# Supplementary material for: Transcriptome analysis suggested that lncRNAs regulate rapeseed seedlings in responding to drought stress by coordinating the phytohormone signal transduction pathways
Source: BMC Genomics. 2024 Jul 19;25:704. doi: 10.1186/s12864-024-10624-4 (PMC11264961; doi:10.1186/s12864-024-10624-4)
Supplement: Supplementary file 7 — Supplementary Material 7 [file 12864_2024_10624_MOESM7_ESM.pdf]

List of primers for quantitative real-time PCR

lncRNA	Sequence (5'-3')	
	Forward primer	Reverse primer
XLOC_000799	TCGTGCTTCCACATCCTCAATA	TGCTGTGATATGAACGAAATTGGG
XLOC_074921	AGCCGTCGCATGATTTGTTG	CGTAGGGGTTAAATCGGGTGT
XLOC_001194	CGATGAACGGTGGAAGAAGC	CCAGAACGTACCTGAACCTGT
XLOC_032712	TCCGGTTAGCGGATTTTGATTC	GAGAAAATGTAGCCAGCGAGC
XLOC_008374	AGCACCTCTTTAATCACGGCT	CGTGTGAGGCAGATCATGGA
XLOC_083471	CGGATTGTACTTTCGCTAGGC	AATTTCCACCGATTCACGCG
XLOC_087430	ACTATCTTCTTCGGTTATGCGC	GGCTTAGCTTCCTCGTTACGA
XLOC_095219	CGGATCCAGATTTGGTGTTCA	CATCTTGAATTCTCCTGCTGGC
XLOC_071711	GGCATTAGTTTCTAGCCGGTT	AAAATCACACGGAATCGGGC
XLOC_071682	TCCGCTAAATCCAGGAAACCT	AAAGCTTAACAGGCCATGGG
