# Supplementary material for: Transcriptome analysis suggested that lncRNAs regulate rapeseed seedlings in responding to drought stress by coordinating the phytohormone signal transduction pathways
Source: BMC Genomics. 2024 Jul 19;25:704. doi: 10.1186/s12864-024-10624-4 (PMC11264961; doi:10.1186/s12864-024-10624-4)
Supplement: Supplementary file 8 — Supplementary Material 8 [file 12864_2024_10624_MOESM8_ESM.pdf]

RNA-seq data for 9 samples

Sample name	Raw reads	Clean reads	Clean bases	Error rate (%)	Q20(%)	Q30(%)	GC content (%)
Q2CK_1	113628736	108435668	16.27G	0.01	98.1	94.92	42.39
Q2CK_2	94658698	90136564	13.52G	0.01	98.2	95.13	42.14
Q2CK_3	97701748	93061914	13.96G	0.01	98.13	94.96	42.39
Q2DS_1	100108630	95342690	14.3G	0.01	98.17	95.08	42.17
Q2DS_2	95403342	91203710	13.68G	0.02	95.69	89.86	42.26
Q2DS_3	88523310	84584352	12.69G	0.02	95.21	88.9	42.15
Q2RW_1	86483028	82369534	12.36G	0.02	95.37	89.2	42.5
Q2RW_2	93887532	89721434	13.46G	0.02	95.38	89.24	42.49
Q2RW_3	89682240	85636906	12.85G	0.02	95.24	88.95	42.32
